# A patatin-like phospholipase is important for mitochondrial function in malaria parasites

**DOI:** 10.1128/mbio.01718-23

**Published:** 2023-10-26

**Authors:** Emma Pietsch, Abhinay Ramaprasad, Sabrina Bielfeld, Yvonne Wohlfarter, Bohumil Maco, Korbinian Niedermüller, Louisa Wilcke, Joachim Kloehn, Markus A. Keller, Dominique Soldati-Favre, Michael J. Blackman, Tim-Wolf Gilberger, Paul-Christian Burda

**Affiliations:** 1 Centre for Structural Systems Biology, Hamburg, Germany; 2 Bernhard Nocht Institute for Tropical Medicine, Hamburg, Germany; 3 University of Hamburg, Hamburg, Germany; 4 Malaria Biochemistry Laboratory, The Francis Crick Institute, London, United Kingdom; 5 Institute of Human Genetics, Medical University of Innsbruck, Innsbruck, Austria; 6 Department of Microbiology and Molecular Medicine, Faculty of Medicine, University of Geneva, Geneva, Switzerland; 7 Faculty of Infectious and Tropical Diseases, London School of Hygiene & Tropical Medicine, London, United Kingdom; Washington University in St. Louis School of Medicine, St. Louis, Missouri, USA

**Keywords:** malaria, mitochondrion, patatin-like phospholipase, electron transport chain, cardiolipin

## Abstract

*Plasmodium* parasites rely on a functional electron transport chain (ETC) within their mitochondrion for proliferation, and compounds targeting mitochondrial functions are validated antimalarials. Here, we localize *Plasmodium falciparum* patatin-like phospholipase 2 (*Pf*PNPLA2, PF3D7_1358000) to the mitochondrion and reveal that disruption of the *Pf*PNPLA2 gene impairs asexual replication. *Pf*PNPLA2-null parasites are hypersensitive to proguanil and inhibitors of the mitochondrial ETC, including atovaquone. In addition, *Pf*PNPLA2-deficient parasites show reduced mitochondrial respiration and reduced mitochondrial membrane potential, indicating that disruption of *Pf*PNPLA2 leads to a defect in the parasite ETC. Lipidomic analysis of the mitochondrial phospholipid cardiolipin (CL) reveals that loss of *Pf*PNPLA2 is associated with a moderate shift toward shorter-chained and more saturated CL species, implying a contribution of *Pf*PNPLA2 to CL remodeling. *Pf*PNPLA2-deficient parasites display profound defects in gametocytogenesis, underlining the importance of a functional mitochondrial ETC during both the asexual and sexual development of the parasite.

**IMPORTANCE:**

For their proliferation within red blood cells, malaria parasites depend on a functional electron transport chain (ETC) within their mitochondrion, which is the target of several antimalarial drugs. Here, we have used gene disruption to identify a patatin-like phospholipase, *Pf*PNPLA2, as important for parasite replication and mitochondrial function in *Plasmodium falciparum*. Parasites lacking *Pf*PNPLA2 show defects in their ETC and become hypersensitive to mitochondrion-targeting drugs. Furthermore, *Pf*PNPLA2-deficient parasites show differences in the composition of their cardiolipins, a unique class of phospholipids with key roles in mitochondrial functions. Finally, we demonstrate that parasites devoid of *Pf*PNPLA2 have a defect in gametocyte maturation, underlining the importance of a functional ETC for parasite transmission to the mosquito vector.

## INTRODUCTION

The protozoan parasite *Plasmodium falciparum* is the major cause of disease and death from malaria, which continues to be one of the most important infectious diseases worldwide (1). After an initial multiplication step in the liver, malaria parasites undergo massive asexual replication within the red blood cells (RBCs) of the human host. In a continuous 48-hour cycle, the blood-stage parasite matures from the ring to the trophozoite and finally to the schizont stage, until the host cell ruptures, releasing up to 32 daughter merozoites to invade new RBCs. The transformation and destruction of RBCs during this stage are responsible for all the clinical symptoms of malaria. During each replication cycle, a small proportion of parasites commit to sexual development and differentiate into gametocytes, which are essential for parasite transmission via the mosquito vector (2).

Despite ongoing efforts to control this persistent infectious disease, progress in reducing the number of deaths and infections from malaria appears to have stalled, in part due to the emergence of parasites resistant to frontline antimalarials (3). Consequently, there is an urgent need to better understand the biology of the malaria parasite in order to identify suitable targets for new intervention strategies. Several antimalarial drugs target the parasite mitochondrion, which is essential for parasite survival and contains both conserved and unique features specific to apicomplexan parasites (4, 5). The mitochondrion grows from a single organelle into a highly branched structure in late asexual blood-stage parasites. These branched structures then undergo ordered fission to be ultimately packed into daughter cell parasites during cytokinesis (6). As in other eukaryotes, the inner membrane of the parasite mitochondrion houses a mitochondrial electron transport chain (ETC). Since asexual blood-stage parasites primarily depend on glycolysis for ATP synthesis, the main function of the ETC is to sustain the essential pyrimidine biosynthesis pathway and maintain the mitochondrial membrane potential (5).

Mitochondrial physiology depends on the phospholipid composition of mitochondrial membranes (7). For instance, a human phospholipase D located in the mitochondrial outer membrane has been implicated in the regulation of mitochondrial fusion (8), and the mitochondrial calcium-independent phospholipase A2γ in mice is important for ETC activity by regulating the metabolism of the mitochondrial phospholipid cardiolipin (CL) (9). However, the importance of phospholipases for mitochondrial function in *Plasmodium* parasites has not been examined.

The *P. falciparum* genome encodes 26 genes with putative phospholipase function. Mass spectrometric evidence for expression in asexual blood stage parasites exists for 19 of these putative enzymes, whose essentiality for asexual replication we previously investigated using a large-scale targeted gene disruption approach (10). Four of these belong to the class of patatin-like phospholipases (PNPLAs), highly conserved enzymes of prokaryotic and eukaryotic organisms with known functions in lipid homeostasis, membrane degradation, and cell signaling, and as important virulence factors in pathogenic bacteria (11). PNPLAs exhibit phospholipase A2 activity and all share a common protein domain with the patatin glycoprotein, a nonspecific lipid acyl hydrolase that is found in high concentrations in mature potato tuber (11, 12). Of the four putative *P. falciparum* PNPLAs, only *Pf*PNPLA1 (PF3D7_0209100) has been examined in greater detail so far. It was shown to be critical for gametocyte induction (13) and gametogenesis (14).

In this study, we characterize a second putative *P. falciparum* PNPLA, here termed *Pf*PNPLA2 (PF3D7_1358000), and show that it has a crucial function in the ETC, which is required for the growth and development of asexual and sexual blood-stage parasites.

## RESULTS

### *Pf*PNPLA2 localizes to the mitochondrion and is important for parasite growth

**Fig 1:**
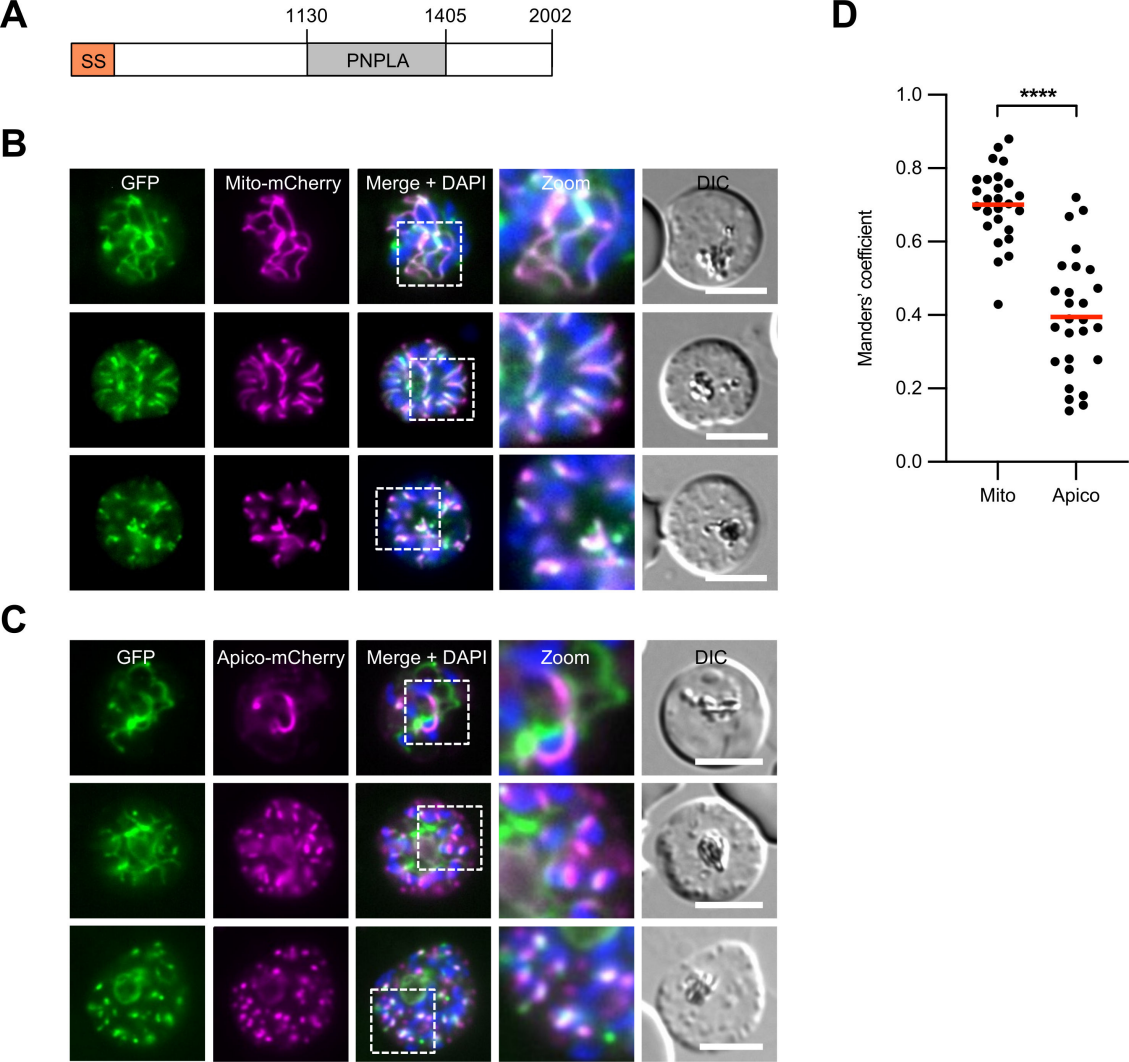
*Pf*PNPLA2 localizes to the mitochondrion. (**A**) Schematic overview of the predicted functional domains of *Pf*PNPLA2. SS, signal sequence; PNPLA, patatin-like phospholipase domain. Amino acid numbers are indicated. (**B and C**) Live-cell microscopy of parasites expressing endogenously tagged *Pf*PNPLA2-GFP (green). Parasites co-expressing the mitochondrial marker Mito-mCherry (magenta) are shown in panel **B**, whereas parasites co-expressing the apicoplast marker Apico-mCherry (magenta) are shown in panel **C**. Merged images additionally contain DAPI-stained nuclei (blue). DIC, differential interference contrast. All scale bars, 5 µm. (**C**) Manders’ coefficients indicative of the fraction of *Pf*PNPLA2-GFP overlapping the mitochondrial and apicoplast markers. Mean values from a total of 26 (Mito-mCherry) and 27 (Apico-mCherry) parasites are highlighted in red. For statistical analysis, unpaired Student’s *t*-test was used (****P < 0.0001).

*Pf*PNPLA2 encodes a protein of 2,012 amino acids with peak expression during schizont development of asexual blood-stage parasites (15). The predicted protein contains an N-terminal signal sequence in addition to a PNPLA domain in the C-terminal half of the protein (residues 1,130–1,405) (Fig. 1A). The N-terminal signal sequence may function as a mitochondrial targeting sequence based on MitoProt II prediction (*P* = 0.9152) (16) or could alternatively function as an apicoplast targeting sequence since *Pf*PNPLA2 was previously predicted to be an apicoplast protein based on all available bioinformatic apicoplast prediction tools (17).

In order to determine the subcellular localization of *Pf*PNPLA2 in the parasite, we appended a C-terminal green fluorescent protein (GFP)-tag to the endogenous gene using the selection-linked integration (SLI) system (18) (Fig. S1A). We confirmed the genetic modification by PCR (Fig. S1B) and analyzed the expression of *Pf*PNPLA2 by live fluorescence microscopy. This showed that *Pf*PNPLA2-GFP predominantly localized to the mitochondrion, confirmed by colocalization of *Pf*PNPLA2-GFP with the mitochondrial marker Mito-mCherry, an mCherry fusion protein that is directed to the mitochondrion via its N-terminal citrate synthase leader sequence (19) (Fig. 1B). Colocalization analysis of *Pf*PNPLA2-GFP and the apicoplast marker Apico-mCherry (20), which relies on the ACP-targeting sequence for its apicoplast localization (21), showed no or only partial colocalization (Fig. 1C). Quantification of colocalization of the organelle-specific markers with *Pf*PNPLA2 by calculating the Manders’ coefficient showed that this was substantially higher for the mitochondrial marker than for the apicoplast marker, further supporting localization of *Pf*PNPLA2 to the parasite mitochondrion (Fig. 1D).

To probe the physiological function of *Pf*PNPLA2 in blood-stage parasite development, we performed targeted gene disruption using the SLI system (Fig. S2A). Hereby, we deleted more than 95% of the *Pf*PNPLA2 coding sequence, including the predicted functional PNPLA domain containing the GXSXG lipase motif, rendering the residual truncated *Pf*PNPLA2 protein enzymatically inactive. The genetic modification was confirmed by PCR (Fig. S2B). In addition to this, we performed western blot analysis of parasites expressing full-length *Pf*PNPLA2-GFP and *Pf*PNPLA2-knockout (KO) parasites expressing truncated *Pf*PNPLA2-GFP, which confirmed the expected sizes for both proteins (267 vs 40 kDa, Fig. S2C). Fluorescence microscopy revealed that the truncated GFP-tagged *Pf*PNPLA2 protein is still localized to the mitochondrion, as evidenced by colocalization with the mitochondrial marker Mito-mCherry (Fig. S2D), indicating that the first 93 amino acids of the protein are enough for targeting the protein to this organelle. Of note, mean fluorescence intensity values were higher for truncated in comparison to full-length *Pf*PNPLA2-GFP (Fig. S2E), which either could indicate higher expression of truncated *Pf*PNPLA2 or may point to the fact that structural changes associated with *P*fPNPLA2-truncation might increase GFP fluorescence intensity.

**Fig 2:**
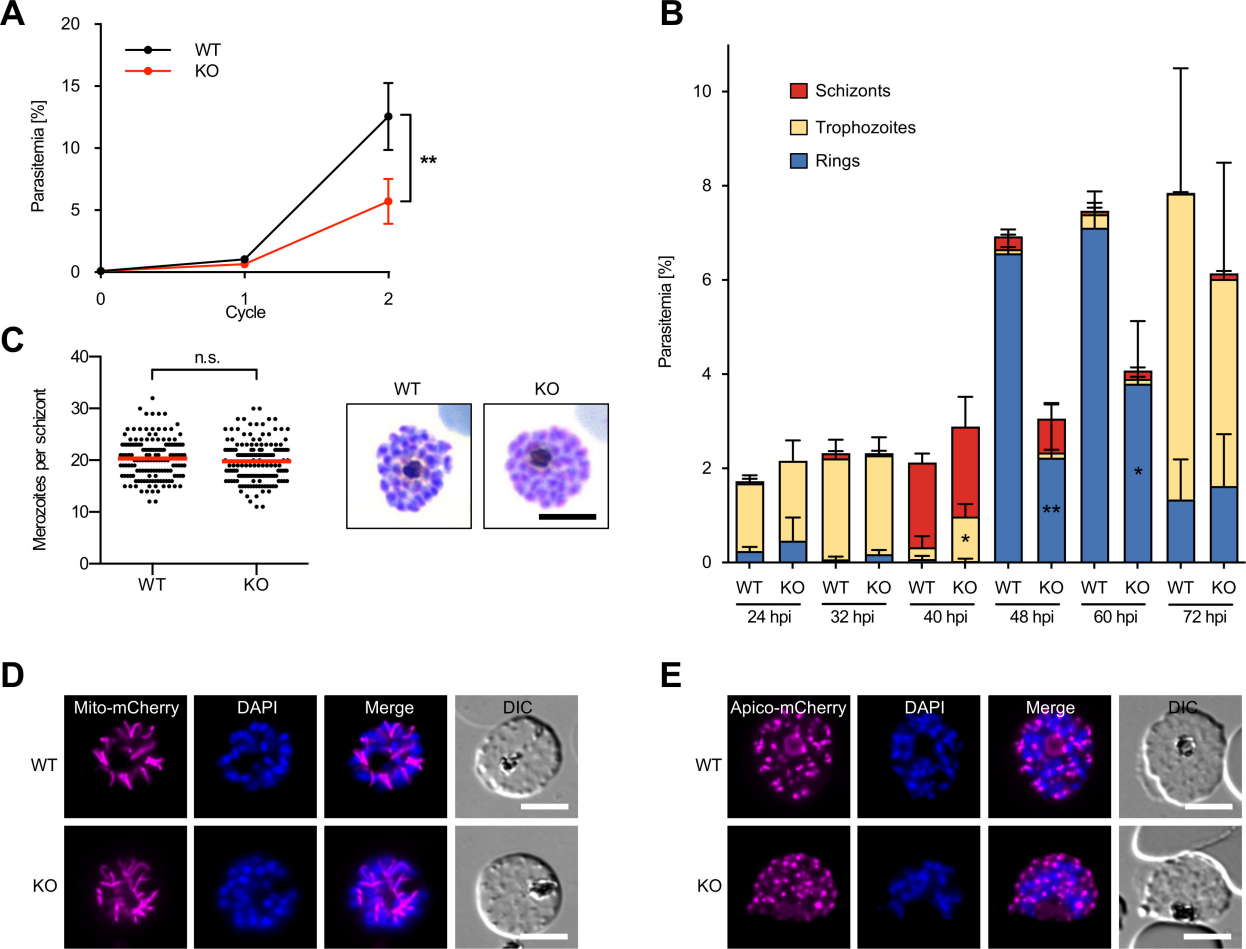
*Pf*PNPLA2 is important for asexual blood-stage proliferation. (**A**) Flow cytometry-based growth analysis of synchronous *Pf*PNPLA2-KO parasite lines over two erythrocytic cycles in comparison to WT parasites. Parasitemia values (means ± SD) of four independent growth experiments are shown. Statistical evaluation of growth data after cycle 2 used unpaired Student’s *t*-test (**P < 0.01). (**B**) Stage quantification of WT and *Pf*PNPLA2-KO parasites at different time points after invasion. Shown are the means ± SD of three independent experiments. Statistical evaluation was performed using unpaired Student’s *t*-test (*P < 0.05 and **P < 0.01). (**C**) Merozoite numbers per segmented schizont in WT and *Pf*PNPLA2-KO parasites. Shown are pooled data from three independent experiments. In each experiment, the number of merozoites per schizont was determined from 50 schizonts per parasite line. Mean values are highlighted in red. Statistical evaluation used unpaired Student’s *t*-test (n.s., not significant). Representative Giemsa-stained images of WT and KO parasites are shown on the right. Scale bar, 5 µm. (**D**) Mitochondrial morphology in WT and *Pf*PNPLA2-KO parasites as visualized by expression of mCherry directed to the mitochondrion (Mito-mCherry, magenta). (**E**) Apicoplast morphology in WT and *Pf*PNPLA2-KO parasites as visualized by the expression of mCherry directed to the apicoplast (Apico-mCherry, magenta). Nuclei were stained with DAPI (blue). DIC, differential interference contrast. All scale bars, 5 µm.

*Pf*PNPLA2-KO parasites consistently showed a reduction in their growth rate of ~50% in comparison to wild-type (WT) parasites over two erythrocytic cycles (Fig. 2A), indicating that *Pf*PNPLA2 is important for normal parasite proliferation. To analyze this phenotype in more detail, we examined the development of tightly synchronized intracellular parasites over the course of the erythrocytic cycle by microscopic quantification of Giemsa-stained thin blood films. *Pf*PNPLA2-KO parasites exhibited delayed development in comparison to WT parasites, as evidenced by a statistically significant increase of KO trophozoite-stage parasites at 40 hours post-infection (hpi) and a significant decrease of newly formed KO ring-stage parasites at both 48 and 60 hpi (Fig. 2B). However, in those *Pf*PNPLA2-KO schizonts that developed to maturity, there was no significant decrease in daughter merozoite numbers (Fig. 2C), indicating that loss of *Pf*PNPLA2 leads to delayed but not compromised parasite maturation. Mitochondrial and apicoplast morphology appeared to be unaffected in *Pf*PNPLA2-KO parasites as determined by episomally expressing mCherry directed to the mitochondrion or apicoplast, respectively (Fig. 2D and E). Examination of *Pf*PNPLA2-null parasites by focused ion beam–scanning electron microscopy (FIB-SEM) identified no obvious morphological differences from WT parasites (Fig. S3; Movies S1 and S2), suggesting that *Pf*PNPLA2 depletion results in delayed parasite maturation without affecting general parasite morphology.

To further analyze the function of *Pf*PNPLA2, we targeted the *pfpnpla2* gene using a DiCre-based conditional KO approach (22, 23). We used Cas9-assisted double homologous recombination to introduce loxP sites flanking the C-terminal half of the *Pf*PNPLA2 coding sequence (harboring the catalytic PNPLA domain) and to simultaneously append a 3xHA epitope tag to the gene (Fig. S4A). This genetic manipulation was performed in the DiCre-expressing B11 *P. falciparum* line (24) and the expected genomic modification was confirmed by PCR (Fig. S5). Rapamycin (RAP) treatment of synchronous ring-stage parasites of the resulting modified parasite line, *Pf*PNPLA2:HA:loxPint, resulted in efficient truncation of the *Pf*PNPLA2 gene within a single erythrocytic cycle, as confirmed by PCR (Fig. S4B) and western blot analysis (Fig. S4C). Flow cytometry-based growth analysis revealed a reduction in replication rate of ~50% in RAP-treated parasites over four erythrocytic cycles, as compared to mock-treated *Pf*PNPLA2:HA:loxPint parasites (Fig. S4D). This growth phenotype was also discernible by plaque assays (25) (Fig. S4E), confirming the SLI-based *Pf*PNPLA2 deletion phenotype using an independent genetic approach. The reduced replication rate of *Pf*PNPLA2-null parasites was not due to inefficient egress or invasion, as no differences in ring-stage parasitemia were observed when isolated schizonts from RAP- and mock-treated *Pf*PNPLA2:HA:loxPint cultures at the end of the cycle of treatment were incubated with fresh RBCs under both static and shaking conditions to allow egress and invasion (Fig. S4F).

### *Pf*PNPLA2-deficient parasites are hypersensitive to drugs targeting mitochondrial function

**Fig 3:**
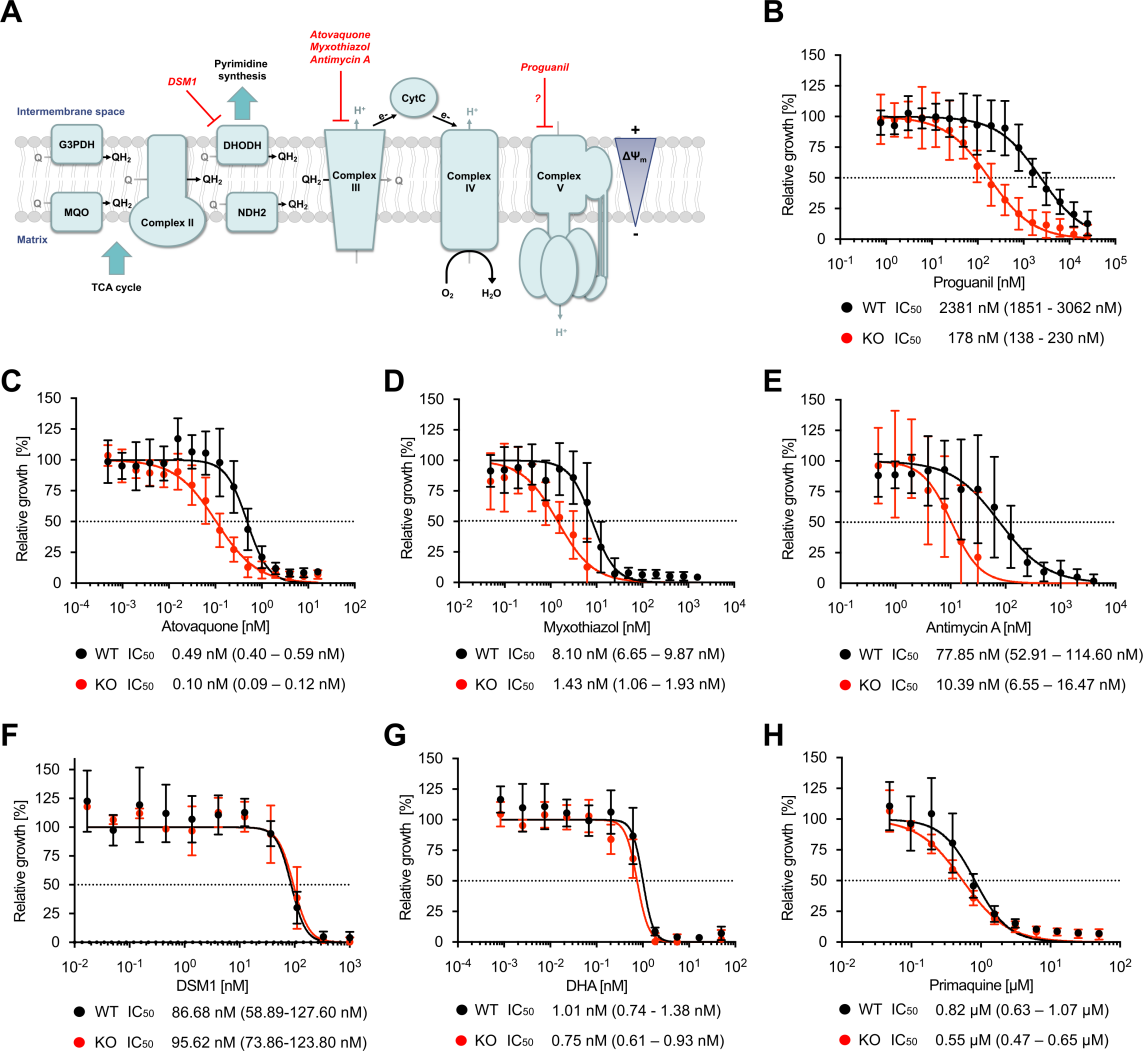
*Pf*PNPLA2-KO parasites are hypersensitive to drugs that target mitochondrial functions. (**A**) Schematic of the ETC in asexual blood-stage parasites of *P. falciparum*. Targets of several drugs targeting mitochondrial functions are highlighted. Ubiquinone (Q), ubiquinol (QH2), glycerol 3-phosphate dehydrogenase (G3PDH), dihydroorotate dehydrogenase (DHODH), malate:quinone oxidoreductase (MQO), type 2 NADH dehydrogenases (NDH2), cytochrome c (CytC), and electrons (e-). (**B–H**) Drug-susceptibility assays of WT and *Pf*PNPLA2-KO parasites using proguanil (**B**), atovaquone (**C**), myxothiazol (**D**), antimycin A (**E**), DSM1 (**F**), dihydroartemisinin (DHA) (**G**), and primaquine (**H**). Parasite growth was assessed by measuring DNA content using SYBR Gold when exposed to varying concentrations of drugs for 96 hours. Growth of DMSO-treated control parasites was set to 100%. Shown are the means ± SD of three to six independent experiments performed in duplicate. Calculated IC_50_ values with 95% confidence intervals are shown below each graph.

Given the predominantly mitochondrial localization of *Pf*PNPLA2, we decided to characterize the susceptibility of our *Pf*PNPLA2-KO parasites to several drugs that target mitochondrial function in order to examine whether depletion of *Pf*PNPLA2 might impact the efficiency of these compounds (Fig. 3A). For this, we performed a 96-hour comparative growth assay starting with trophozoite-stage parasites cultured in the presence of varying concentrations of the drugs. In these and all the following experiments, we made use of SLI-based *Pf*PNPLA2-KO parasites. In comparison to WT parasites, *Pf*PNPLA2-null parasites showed decreased IC_50_ values for proguanil (~13-fold) and the ETC inhibitors atovaquone (approximately fivefold), myxothiazol (approximately sixfold), and antimycin A (approximately sevenfold) (Fig. 3B through E). The *Pf*PNPLA2-null parasites showed no increased sensitivity to the dihydroorotate dehydrogenase (DHODH) inhibitor DSM1 (26), or drugs targeting non-mitochondrial processes such as dihydroartemisinin (DHA) and primaquine (Fig. 3F through H), excluding a general increased drug susceptibility of the *Pf*PNPLA2-KO parasites. Collectively, these data suggest that disruption of *Pf*PNPLA2 sensitizes parasites to antimalarial drugs that inhibit mitochondrial function.

### Disruption of *Pf*PNPLA2 impairs the function of the ETC

**Fig 4:**
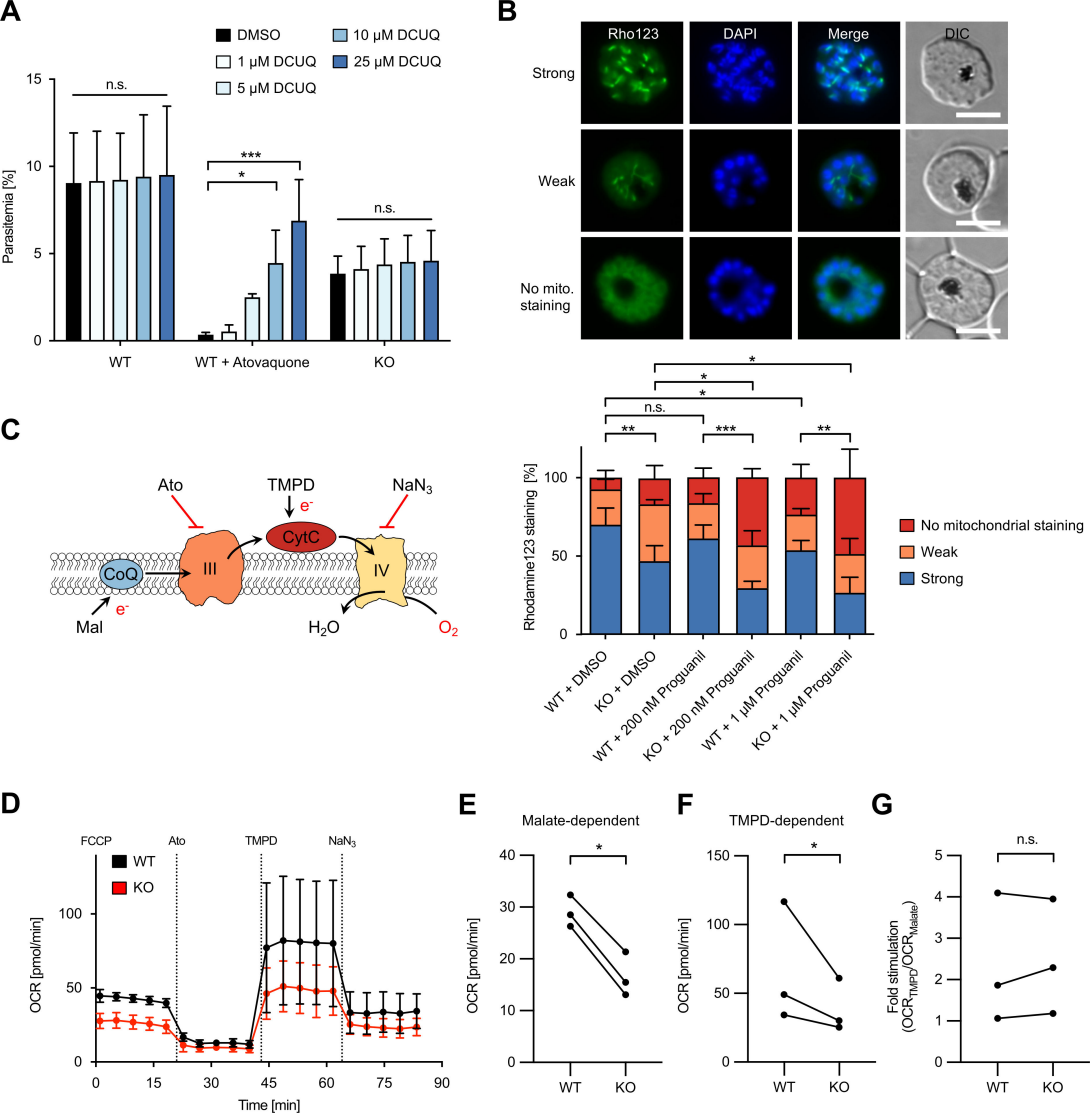
*Pf*PNPLA2 is important for a functional ETC likely downstream of cytochrome c. (**A**) The artificial electron acceptor DCUQ does not rescue the growth of *Pf*PNPLA2-KO parasites. WT and *Pf*PNPLA2-KO parasites were grown in the presence of various concentrations of DCUQ for two erythrocytic cycles and parasitemia was evaluated using flow cytometry. As a positive control, WT parasites were additionally treated with 1.15 nM atovaquone. Shown are the means + SD of three independent experiments. Statistical evaluation was performed using ANOVA followed by a Holm-Sidak multiple comparison (*P < 0.05; ***P < 0.001; and n.s.; not significant). (**B**) Analysis of the mitochondrial membrane potential ΔΨ_m_ in WT and *Pf*PNPLA2-KO parasites. C2-arrested WT and *Pf*PNPLA2-KO schizonts that had been treated with DMSO (solvent control) or proguanil (at 200 nM or 1 µM) were stained with the mitochondrial potentiometric dye rhodamine123 (Rho123, green) and parasites with a strong, weak or no mitochondrial rhodamine123 signal were quantified by fluorescence microscopy. Shown are the means ± SD of four independent experiments, in which a total of 352–414 schizonts were analyzed per cell line and condition. For statistical evaluation of the percentage of parasites showing an abnormal rhodamine123 signal (weak or no mitochondrial staining), a one-way ANOVA followed by a Holm-Sidak multiple comparison test was performed (*P < 0.05; **P < 0.01; ***P < 0.001; and n.s.; not significant). Representative images are shown on top of the graph. DAPI-stained nuclei are shown in blue. DIC, differential interference contrast. Scale bars, 5 µm. (**C**) Schematic of the assay measuring the OCR of permeabilized, FCCP-treated *P. falciparum* parasites supplied with malate (Mal) as a substrate to which atovaquone, TMPD/ascorbate, and sodium azide (NaN_3_) were sequentially added. CoQ, coenzyme Q (ubiquinone); III, Complex III; CytC, cytochrome c; IV, Complex IV; and e-, electrons. (**D**) Traces depicting parasite OCR over time. Shown are the means ± SD of three independent experiments. (**E–G**) Malate-elicited OCR after FCCP treatment (**E**), TMPD-induced OCR (**F**), and fold stimulation of OCR by TMPD relative to malate in *Pf*PNPLA2-KO in comparison to WT parasites (**G**). Shown are the results of three independent experiments. For statistical analyses, a ratio-paired Student’s *t*-test was performed (*P < 0.05 and n.s., not significant).

One main role of the malarial ETC is to recycle ubiquinone, which is necessary for ubiquinone-dependent enzymes. These include DHODH, which is essential for the pyrimidine biosynthesis pathway (Fig. 3A) (4, 26). To test whether the ubiquinone pool was affected by the disruption of *Pf*PNPLA2, we treated parasites with increasing concentrations of the ubiquinone analog decylubiquinone (DCUQ). As reported previously (27), this treatment rescued an atovaquone-induced growth arrest of WT parasites in a dose-dependent manner. However, the growth phenotype of *Pf*PNPLA2-KO parasites could not be rescued (Fig. 4A). This together with the comparable susceptibility of *Pf*PNPLA2-KO and WT parasites toward DSM1 suggests that disruption of *Pf*PNPLA2 likely does not impair ubiquinone recycling.

Apart from ubiquinone recycling, another important function of the ETC in blood-stage malaria parasites is to pump protons across the mitochondrial inner membrane to generate a proton electrochemical gradient. The electrical component of the resulting proton motive force is the mitochondrial membrane potential (ΔΨ_m_). The energy saved in the ΔΨ_m_ is used by the mitochondrion to import proteins and metabolic precursors from the cytosol, to maintain critical biochemical pathways such as the generation of iron-sulfur clusters (28, 29). To test whether the ΔΨ_m_ is affected in *Pf*PNPLA2-KO parasites, we incubated WT and *Pf*PNPLA2-KO schizonts in the presence of the mitochondrial potentiometric dye rhodamine123 as previously described (30). Subsequently, parasites were analyzed by fluorescence microscopy. Three different staining patterns were identified with parasites displaying either: (i) a strong punctate mitochondrial rhodamine123 signal; (ii) a weak but clearly punctate mitochondrial staining just above the background; or (iii) no mitochondrial staining (cytoplasmic/peripheral rhodamine123 signal or no fluorescence signal at all). As shown in Fig. 4B, significantly more *Pf*PNPLA2-KO parasites showed an abnormal (weak or no mitochondrial) rhodamine123 signal in comparison to WT parasites, indicating that disruption of *Pf*PNPLA2 impairs a mitochondrial function that is necessary to sustain a normal ΔΨ_m_.

Given that, of the drugs tested, *Pf*PNPLA2-KO parasites showed the highest level of hypersensitivity toward proguanil (Fig. 3B), parasites were also treated with increasing concentrations of this drug to test how this affected the ΔΨ_m_. In line with our drug susceptibility data, a concentration of 200 nM proguanil led to a statistically significant increase in *Pf*PNPLA2-KO parasites showing an abnormal rhodamine123 staining in comparison to dimethyl sulfoxide (DMSO)-treated parasites, while the differences observed for WT parasites at this concentration of the drug did not reach statistical significance (Fig. 4B). Of note, treating WT parasites with 1 µM proguanil also led to a small yet significant increase of the percentage of parasites showing an abnormal rhodamine123 signal, suggesting that proguanil treatment alone impacts on the ΔΨ_m_ in WT parasites. Collectively, our data imply that *Pf*PNPLA2-KO parasites may have a defect in the ETC, which leads to an impaired ability to sustain the ΔΨ_m_ and thereby renders the parasite hypersensitive to ETC inhibitors.

### The *Pf*PNPLA2-associated defect in the ETC likely occurs downstream of cytochrome c

To further characterize the *Pf*PNPLA2-dependent defect in the ETC and to pinpoint where it might be occurring, we aimed to analyze the oxygen consumption rate (OCR) at Complex IV in *Pf*PNPLA2-deficient parasites. The OCR is an indicator of the electron flow through the ETC and can be used to bioenergetically assess the ETC enzymes and to characterize mitochondrial dysfunction (Fig. 4C) (31). To measure the OCR, we subjected WT and *Pf*PNPLA2-KO schizonts to an XFe96 flux analyzer-based assay, previously adapted for *P. falciparum* and the related apicomplexan parasite *Toxoplasma gondii* (31–33). To this end, we released parasites from host RBCs using saponin and gently permeabilized the plasma membranes of the free parasites using digitonin in the presence of malate, which feeds electrons into the ETC via the TCA cycle enzyme malate:quinone oxidoreductase. We then injected the protonophore carbonyl cyanide-p-trifluoromethoxyphenylhydrazone (FCCP) to induce oxygen consumption at maximum capacity. This demonstrated that the resulting OCR was ~40% reduced in *Pf*PNPLA2-KO parasites in comparison to WT parasites (Fig. 4D and E), further supporting our previous results that disruption of *Pf*PNPLA2 leads to a partial defect in the ETC. As expected, subsequent injection of the Complex III inhibitor atovaquone almost completely abolished OCR levels, confirming that the observed OCR indeed represents mitochondrial respiration (Fig. 4D).

We next wondered whether the defect in the malate-dependent OCR upon *Pf*PNPLA2 deletion occurred upstream or downstream of cytochrome c. To analyze this, we made use of the cytochrome c substrate N, N, N′, N′-tetramethyl-p-phenylenediamine dihydrochloride (TMPD), as previously performed in *T. gondii* (32). Reduced TMPD donates electrons directly to cytochrome c, bypassing Complex III, and is, therefore, expected to rescue OCR in parasites with an ETC defect upstream of cytochrome c, while a defect downstream of cytochrome c at Complex IV should not be rescued by TMPD (Fig. 4C). Injection of TMPD caused an increase in the OCR of both WT and *Pf*PNPLA2-KO parasites, indicating that Complex IV is at least partially active in both parasite lines. This TMPD-induced OCR was substantially decreased upon injection of the Complex IV inhibitor sodium azide (NaN_3_), validating that it was a result of Complex IV activity (Fig. 4D). Importantly, however, a reduced OCR in *Pf*PNPLA2-KO parasites was still detected after TMPD treatment and the fold stimulation of OCR by TMPD relative to malate-dependent OCR was comparable between WT- and *Pf*PNPLA2-deficient parasites (Fig. 4D, F, and G). This demonstrates that a partial defect in the ETC of *Pf*PNPLA2-KO parasites likely occurs downstream of cytochrome c in electron transport from the latter to Complex IV or in activity of Complex IV itself.

### *Pf*PNPLA2 deletion affects mitochondrial cardiolipin metabolism

Given that *Pf*PNPLA2 is annotated as a phospholipase and has a conserved PNPLA domain containing the characteristic GXSXG lipase motif (11), we next sought to further understand the *Pf*PNPLA2-deletion phenotype by targeted lipidomic analysis. We focused on the mitochondrion-specific phospholipid cardiolipin, due to its established key role in mitochondrial functions and the ETC (34–36). CL is a phospholipid with two phosphate headgroups and four fatty acyl chains. Its *de novo* synthesis in the mitochondrion is followed by a unique remodeling process, which is driven by the activity of the transacylase tafazzin, but can also be initiated by a phospholipase-catalyzed side-chain hydrolysis to form monolyso-cardiolipin (MLCL, CL lacking one acyl chain) and completed by a transacylase- or an acyltransferase-dependent reacylation (37). A specific fatty acyl composition is generated during this process, and remodeled CL typically contains predominantly unsaturated fatty acids with longer chain lengths (37). The exact mitochondrial CL profile that can be achieved through remodeling is dependent on the fatty acid and phospholipid state within the cell (38, 39) and the lipid environment outside the cell (40). Apart from CL, we additionally analyzed the levels of several other highly abundant phospholipids, including phosphatidylethanolamine (PE) and phosphatidylglycerol (PG) species, given that both phospholipids have been demonstrated in other systems to influence ETC activity as well (41–43).

**Fig 5:**
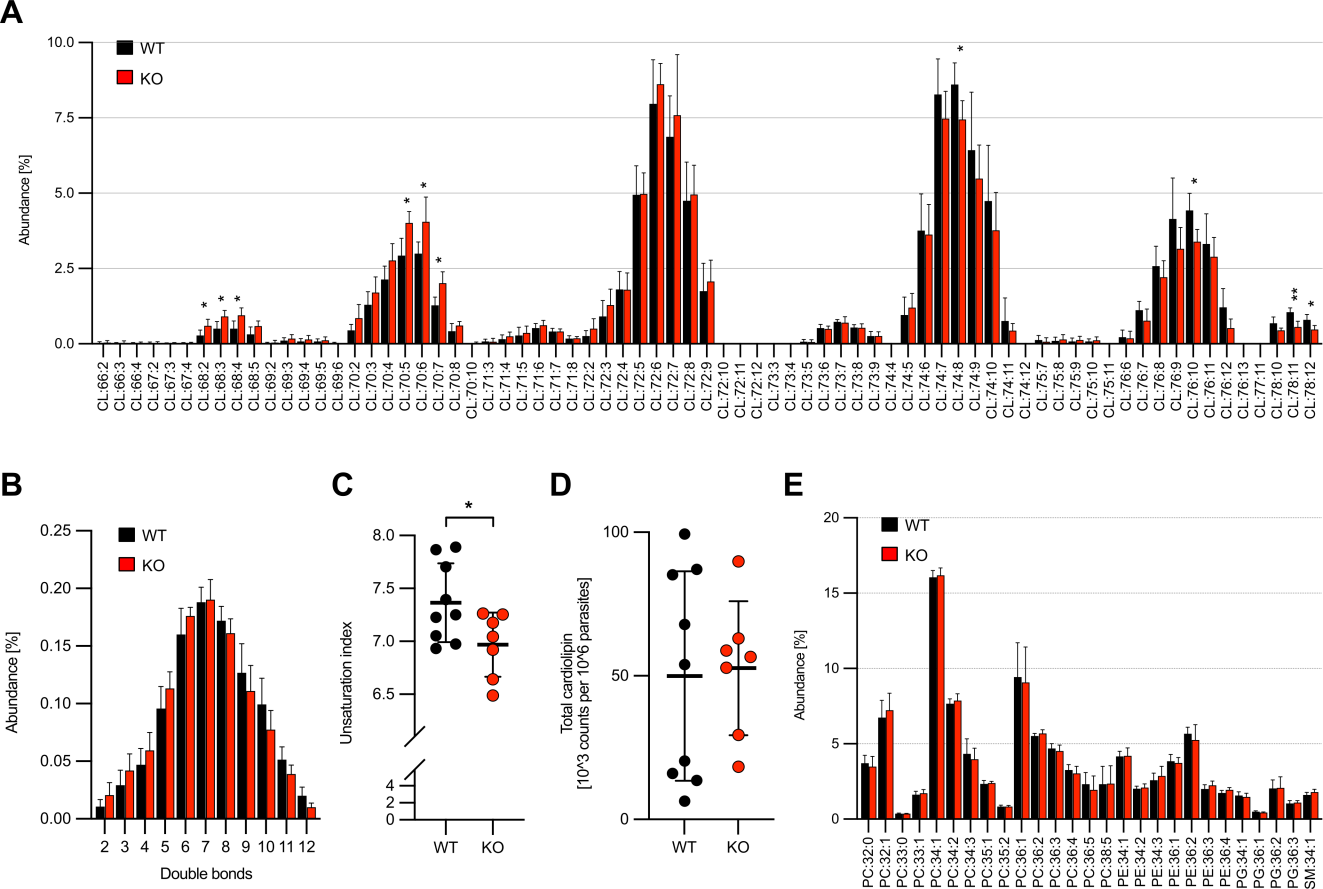
*Pf*PNPLA2 deletion affects mitochondrial CL metabolism. (**A**) CL species distribution and relative abundance in WT and *Pf*PNPLA2-KO schizonts. CL species are sorted according to their total carbon chain length and their total number of double bonds. Shorter species are displayed on the left and longer species on the right. Nomenclature: CL:(number of side chain carbons):(number of side chain double bonds). (**B**) Average abundance-weighted number of double bonds in CL species. (**C**) Unsaturation index of CL species. (**D**) Total CL levels. (**E**) Relative abundance of non-CL lipids. Shown are the means ± SD of seven to nine independent biological replicates. Statistical analysis of the results was done using unpaired Student’s *t*-test. For the multiple *t*-tests in A, B, and E, FDR adjustments (at 5% using the Benjamini-Krieger-Yekutieli procedure) were performed and adjusted *P*-values are shown. Statistically significant differences are highlighted (*P < 0.05 and **P < 0.01). Source data of this figure can be found in File S1.

The lipidomic analysis first revealed that (i) CL species in the malaria parasite are unusually long-chained as compared to other organisms (44); (ii) the CL profile follows the usual pattern generated by predominantly even-numbered side-chain fatty acids (Fig. 5A); and (iii) the parasite CLs show a broad structural diversity, especially in regard to the variability in the total double bond number (Fig. 5B). Comparative analysis of CL composition between *Pf*PNPLA2-KO and WT parasites uncovered shorter and more saturated CL species in *Pf*PNPLA2-deficient parasites (Fig. 5A and B). To further statistically evaluate this, we also calculated the CL unsaturation index, which is the weighted average of the double bond number in CLs (45, 46). This showed that *Pf*PNPLA2-KO parasites had a significantly lower CL unsaturation index as compared to WT parasites (Fig. 5C). Interestingly, the total amount of CLs in parasites was not changed upon *Pf*PNPLA2 deletion (Fig. 5D), suggesting that *Pf*PNPLA2 functions to affect CL composition, but not its abundance. Of note, our analysis did not reveal any major changes in the profile of the investigated non-CL phospholipid species (Fig. 5E) and in their number of double bonds (Fig. S6).

### *Pf*PNPLA2 is important for sexual blood-stage development

**Fig 6:**
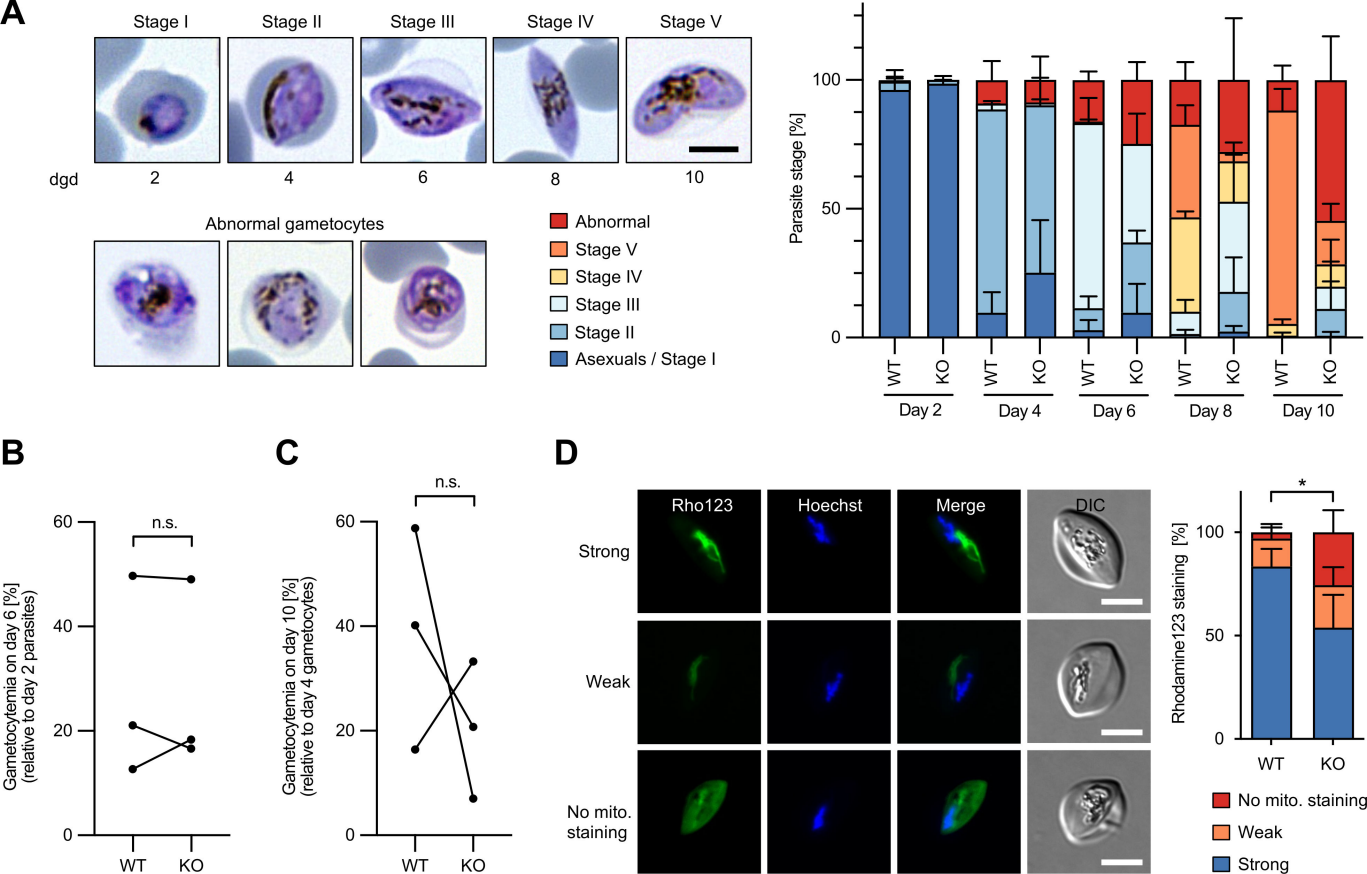
*Pf*PNPLA2 is important for sexual blood-stage development. (**A**) *Pf*PNPLA2-deficient parasites show impaired gametocyte maturation. Quantification of gametocyte stages over 10 days of gametocyte development (dgd). Representative Giemsa-stained gametocytes are shown on the left. Scale bar, 5 µm. Shown are the means ± SD of three independent experiments. (**B and C**) Sexual conversion rate (**B**) and gametocyte survival rate (**C**) of WT and *Pf*PNPLA2-KO parasites. Values of three independent experiments are shown. Statistical analysis used unpaired Student’s *t*-test (n.s., not significant). (**D**) Analysis of the mitochondrial membrane potential ΔΨ_m_ in WT and *Pf*PNPLA2-KO gametocytes at day 6 of gametocyte development. Gametocytes were stained with the mitochondrial potentiometric dye rhodamine123 (Rho123, green), and gametocytes with a strong, weak, or no mitochondrial rhodamine123 signal were quantified by fluorescence microscopy. Shown are the means ± SD of four independent experiments, in which a total of 146 WT and 106 KO gametocytes were analyzed. For the statistical evaluation of the percentage of gametocytes showing an abnormal rhodamine123 signal (weak or no mitochondrial staining), an unpaired Student’s *t*-test was performed (*P < 0.05). Representative images are shown on the left. Hoechst-stained nuclei are shown in blue. DIC, differential interference contrast. Scale bars, 5 µm.

Our results indicate an important function of *Pf*PNPLA2 in mitochondrial lipid homeostasis and in the ETC during the asexual development of the parasite within RBCs. Complexes of the ETC and linked metabolic pathways were previously shown to be up to 40-fold more prevalent in gametocytes, the sexual stages of the parasite (47). Other earlier work has shown that inhibitors of the ETC affected not only the asexual but also the sexual stages of the parasite (48, 49). Thus, we next explored the function of *Pf*PNPLA2 in sexual blood-stage development. For this purpose, we performed SLI-based targeted gene disruption of *Pf*PNPLA2 in a 3D7-derived parasite line that fully supports gametocyte development (50). We verified the correct integration of the targeting plasmid into the *pfpnpla2* locus by PCR (Fig. S7A and B) and confirmed the expected KO-associated growth defect of this parasite line during asexual blood-stage development (Fig. S7C). We then induced gametocyte formation by the removal of choline from the medium and depleted residual asexual blood-stage parasites by the addition of N-acetyl-D-glucosamine (GlcNac) from day 1 until day 6 of gametocyte development as previously described (50). WT parasites developed normally from stage I to stage V gametocytes within 10 days after induction. During the first days, *Pf*PNPLA2-KO parasites showed similar yet delayed development, maturing to mostly stage III gametocytes by day 6. Thereafter, the majority of KO gametocytes arrested in maturation and/or displayed an abnormal morphology, indicating that *Pf*PNPLA2 disruption impairs gametocyte development (Fig. 6A). As a measure of sexual conversion, we compared the gametocytemia values from day 6 of gametocyte development to parasitemia values from day 2 of gametocyte development, at which both sexual and asexual blood stages were still present. This did not reveal any differences between WT and KO parasites (Fig. 6B). Gametocyte survival over time, as quantified by dividing gametocytemia values from day 10 by those from day 4, also did not statistically differ between WT and KO parasites (Fig. 6C), underlining the principal importance of *Pf*PNPLA2 for gametocyte maturation. To quantify the mitochondrial membrane potential ΔΨ_m_, we stained WT- and *Pf*PNPLA2-deficient gametocytes with rhodamine123 at day 6 of gametocyte development, where no major differences in the number of gametocytes displaying an abnormal morphology were visible between WT and KO parasites (Fig. 6A). Similar to asexual blood stages (Fig. 4B), a higher percentage of *Pf*PNPLA2-KO gametocytes showed an abnormal rhodamine123 staining (weak or no mitochondrial staining) in comparison to WT parasites (Fig. 6D). Of note, no obvious differences in parasite morphology between parasites showing a normal or an abnormal rhodamine123 staining were observed (Fig. S7D), all in all suggesting that the partial loss of membrane potential may be rather one of the causes than the consequence of the abnormal gametocyte morphology observed for *Pf*PNPLA2-KO parasites at later stages of gametocyte development. Altogether, these data underline that *Pf*PNPLA2 is important for normal ETC function not only in asexual blood stages but also in gametocytes.

## DISCUSSION

The single mitochondrion of malaria parasites is an essential organelle and the target of several antimalarial drugs. Here, we have shown that the malaria parasite *P. falciparum* depends on the putative phospholipase *Pf*PNPLA2 for normal mitochondrial function and that loss of *Pf*PNPLA2 impairs the growth and development of both asexual and sexual blood-stage parasites.

Our observation that *Pf*PNPLA2 localizes to the parasite mitochondrion supports previous detection of the protein in the proteome of the parasite mitochondrion (51). Interestingly, the putative ortholog of *Pf*PNPLA2 in the related apicomplexan parasite *T. gondii* (TGME49_231370) localizes to the apicoplast and its knockdown led to a rapid apicoplast loss due to deregulated lipid homeostasis in this organelle (52). This indicates surprising differences in the functions of putative phospholipase orthologs between these two apicomplexan parasites.

Disruption of *Pf*PNPLA2 impairs asexual blood-stage proliferation and *Pf*PNPLA2-deficient parasites are delayed in their maturation. This phenotype is probably attributable to mitochondrial disfunction since *Pf*PNPLA2-KO parasites (i) are hypersensitive to drugs that target the ETC; (ii) have a defect in sustaining the ΔΨ_m_; and (iii) have a reduced malate-dependent OCR. Given the fact that we were not able to rescue the reduced OCR by TMPD treatment (which directly donates electrons to cytochrome c), we conclude that the defect in the ETC likely occurs downstream of cytochrome c either in electron transport from the latter to Complex IV or in activity of Complex IV itself. This is supported by no measurable hypersensitivity toward DSM1 and no rescue of the *Pf*PNPLA2-KO-associated growth defect by treatment with the ubiquinone analog DCUQ despite its ability to restore a growth defect induced by the Complex III inhibitor atovaquone. In this regard, it is interesting to note that certain phospholipids of the inner mitochondrial membrane, in particular CL and PE, are important for the full activity of the ETC and the efficient generation of ΔΨ_m_ by affecting supercomplex-formation between Complex III and IV and by regulating Complex IV activity in yeast cells (43, 53).

Given the established key role of CL in mitochondrial functions and the known importance of different phospholipases for CL remodeling (37), we performed a lipidomic analysis of CL composition in *Pf*PNPLA2-deficient parasites; to our knowledge, this represents the first detailed characterization of this lipid in *Plasmodium*. This showed that loss of *Pf*PNPLA2 is associated with a moderate increase in CL species with shorter and more saturated acyl groups, while CL abundance is unaffected, suggesting that loss of *Pf*PNPLA2 does not impair CL biosynthesis but rather CL remodeling. A similar phenotype was previously described in yeast, where deletion of the CL-specific mitochondrial phospholipase CLD1 increased acyl saturation without affecting CL abundance (54). It is also possible that the here-observed CL profile changes are caused indirectly, as a consequence of the strong dependence of CL composition on general phospholipid metabolism (37–39), as well as its interplay with the extracellular lipid environment (40). However, the effects of the *Pf*PNPLA2-KO on fatty acid length and saturation were primarily detectable in CLs, but not in other phospholipid species. Given that CL is a mitochondria-exclusive lipid, we also cannot completely exclude that the observed differences in CL composition are the result of mitochondrial functional changes associated with *Pf*PNPLA2 disruption.

In *Drosophila* and mammalian cells, the calcium-independent phospholipases iPLA2β or iPLA2γ have been implicated in CL remodeling (55, 56). Ablation of the mitochondrial iPLA2γ in mice was associated with deregulated CL metabolism and reduced Complex IV activity (9). A previous bioinformatic analysis predicted *Pf*PNPLA2 to be the iPLA2γ homolog in *P. falciparum* and to be involved in CL remodeling (57). The same study did not identify enzymes that are responsible for the reacylation of MLCL (acyl-CoA:lysocardiolipin acyltransferase 1 or tafazzin) in the phylogenetic clade of Alveolata (including *P. falciparum*), leading to the question of how this biochemical reaction is enzymatically realized in these organisms. If *Pf*PNPLA2 is indeed the *P. falciparum* homolog of the iPLA2γ involved in CL remodeling, we may speculate that this function impacts mitochondrial physiology and could explain the observed *Pf*PNPLA2 deletion phenotype. CL acyl chain composition can influence membrane properties in biomimetic membranes, suggesting that it may also impact mitochondrial inner membrane biophysical organization and function (58). Moreover, sequestration of CLs in protein supercomplexes and binding of CL to cytochrome c are promoted by unsaturated CL species (59, 60), and both likely affect ETC function.

Examination of the susceptibility of *Pf*PNPLA2-KO parasites to drugs that inhibit mitochondrial functions revealed that in addition to ETC inhibitors, *Pf*PNPLA2-KO parasites also became hypersensitive toward proguanil, which is used in combination with atovaquone in the registered antimalarial formulation Malarone. Although the mechanism of action of proguanil is not completely understood, combination studies have shown that proguanil acts by lowering the concentration at which atovaquone collapses the parasite ΔΨ_m_ (28, 61). It is hypothesized that this process is connected to an ATP synthase function, which only becomes essential when the ETC is inhibited. During ETC inhibition, ATP synthase could maintain the ΔΨ_m_ by operating in reverse and it may be this function that is inhibited by proguanil (28). Given the high level of hypersensitivity of *Pf*PNPLA2-deficient parasites toward proguanil, it is tempting to speculate that the proguanil-sensitive pathway for maintaining ΔΨ_m_ in the presence of ETC inhibition might partially compensate for the *Pf*PNPLA2-KO-associated defect in the ETC and that this is one reason why *Pf*PNPLA2-KO parasites are still viable.

Similar to our findings in asexual blood stages, ablation of *Pf*PNPLA2 resulted in a defect in gametocyte maturation and an impaired ability of gametocytes to sustain the mitochondrial membrane potential, implicating *Pf*PNPLA2 in ETC function also during sexual development of the parasite. The majority of *Pf*PNPLA2-deficient parasites developed only to stage III gametocytes, thereafter displaying abnormal morphology, and only a small percentage of KO-parasites formed stage V gametocytes, a phenotype that likely strongly impairs subsequent transmission to the mosquito vector. This stands in contrast to the *Pf*PNPLA2-null phenotype in asexual blood-stage parasites, where delayed development was not associated with any gross morphological defects. Although we cannot exclude at this point that *Pf*PNPLA2 is involved in function(s) other than maintaining a functional ETC, these results would argue for a higher dependence of gametocytes on a functional ETC in comparison to asexual blood-stage parasites. This would be in line with several previous studies indicating that mitochondrial functions are more important in the sexual development of malaria parasites than during asexual proliferation (47, 62–64).

In conclusion, our study reveals the importance of *Pf*PNPLA2 for mitochondrial function in both asexual blood stages and gametocytes of *P. falciparum*, highlighting mitochondrial lipid metabolism as a potential target for future drugs with activity against transmission and disease-causing blood stages.

## MATERIALS AND METHODS

### *P. falciparum* parasites

Blood stages of 3D7 *P. falciparum* parasites and all transgenic derivates were cultured in human RBCs. Cultures were maintained at 37°C in an atmosphere of 90% nitrogen, 5% carbon dioxide, and 5% oxygen (DiCre-based KO line) or in an atmosphere of 94% nitrogen, 5% carbon dioxide, and 1% oxygen (all other parasite lines) using RPMI complete medium containing 0.5% Albumax according to standard procedures (65).

### Cloning of plasmids

For the generation of the SLI-based *Pf*PNPLA2 GFP-tagging construct, the C-terminal 1,063 bp of the *pnpla2* gene were amplified by PCR using primers PF3D7_1358000-tag-fw/PF3D7_1358000-tag-rev and cloned into pSLI-TGD (18) using NotI/MluI. For the generation of the *Pf*PNPLA2 TGD construct, 532 bp immediately downstream of the start ATG of the *pnpla2* gene were amplified by PCR using primers PF3D7_1358000-TGD-fw/PF3D7_1358000-TGD-rev and cloned using NotI/MluI into pSLI-TGD. For cloning of the episomal mitochondrial marker plasmid pNMD3:Mito-mCherry-DHODH, the citrate synthase leader sequence (19) was amplified from genomic DNA using primers CytSyn-fw/Cyt-Syn-rev and cloned via XhoI/KpnI into pNMD3:PfLCN-mCherry-DHODH (66), thereby replacing the *Pf*LCN coding sequence.

For the generation of conditional *Pf*PNPLA2-KO parasites, a 2,352 bp long gene segment containing the catalytic domain of the *pfpnpla2* gene was replaced by a synthetic, modified version using Cas9-enhanced homologous recombination by transfecting a guide plasmid and a linearized repair plasmid into the DiCre-expressing *P. falciparum* line B11 (24). Two single guide RNA (sgRNA) inserts were generated by annealing oligo pairs PF3D7_1358000_gRNA01.F/PF3D7_1358000_gRNA01.R and PF3D7_1358000_gRNA02.F/PF3D7_1358000_gRNA02.R, which were subsequently ligated into the BbsI-digested plasmid pDC2-Cas9-hDHFRyFCU plasmid (67), which contains sequences encoding Cas9, single guide RNA (sgRNA), and the drug-selectable marker hDHFR (human dihydrofolate reductase)/yFCU (yeast cytosine deaminase/uridyl phosphoribosyl transferase). The repair plasmid was designed such that it had (i) ~500 bp native sequence on either side of the targeted gene segment to serve as homology arms, (ii) a short synthetic intron containing a *loxP* site (loxPint) upstream of the targeted gene segment, (iii) the recodonized version of the targeted gene segment with the PAM sites destroyed, (iv) a triple-hemagglutinin (3HA) epitope tag just prior to the gene translational stop codon, and (v) another *loxP* site following the translational stop codon. The designed construct was synthesized as two parts (2,421 and 1,109 bp) and inserted subsequently into a pCR-blunt vector (Thermo Fisher Scientific, Waltham, MA, USA) using restriction-ligation with XhoI/ApaI and XhoI/PstI, respectively. The synthesized construct did not contain the 3′ homology arm, which was, therefore, amplified from B11 genomic DNA (amplification primers: PF3D7_1358000_3hom_F and PF3D7_1358000_3hom_R) and added to the construct by restriction-ligation using NheI/ApaI sites to create pREP-*Pf*PNPLA2-3HA-loxPint. Synthetic gene constructs were synthesized by GeneArt (Thermo Fisher Scientific, Waltham, MA, USA).

Phusion High-Fidelity DNA polymerase (New England BioLabs, Ipswich, MA, USA) was used for all plasmid constructions and all plasmid sequences were confirmed by Sanger sequencing. For sequences of all primers and synthetic gene constructs, see File S2.

### Generation of SLI-based parasite lines

For the transfection of constructs, Percoll (GE Healthcare, Chicago, IL, USA)-enriched synchronized mature schizonts of 3D7 parasites were electroporated with 50 µg of plasmid DNA using a Lonza Nucleofector II device (68). Transfectants were selected in medium supplemented with 3 nM WR99210 (Jacobus Pharmaceuticals, Princeton, NJ, USA). For the generation of stable integrant cell lines, parasites containing the episomal plasmids selected with WR99210 were grown with 400 µg/mL neomycin/G418 (Sigma, St. Louis, MO, USA) to select for integrants carrying the desired genomic modification as described previously (18). Successful integration was confirmed by diagnostic PCR using FIREpol DNA polymerase (Solis BioDyne, Tartu, Estonia). For primer sequences, see File S2. For the visualization of the mitochondrion and the apicoplast, parasites were transfected with pNMD3:Mito-mCherry-DHODH and pACP-mCherry_CRT:BSD (20) and selected with 0.9 µM DSM1 (BEI Resources, NIAID, NIH) or 2 µg/mL blasticidin S (Invitrogen, Waltham, MA, USA), respectively.

### Generation of conditional *Pf*PNPLA2-KO parasites

The transgenic *P. falciparum* DiCre-based *Pf*PNPLA2-KO parasite line generated in this study was based on the DiCre-expressing *P. falciparum* clone B11, derived from the 3D7 parasite line (24). Two transfections (one per guide RNA) were performed. Mature schizonts enriched using Percoll (GE Healthcare, Chicago, IL, USA) were electroporated with 20 µg of guide plasmid and 60 µg of linearized repair plasmid using an Amaxa 4D electroporator and P3 Primary cell 4D Nucleofector X Kit L (Lonza, Basel, Switzerland) using program FP158 as described (22). At 24 hours post-transfection, the culture medium was replaced with fresh medium containing WR99210 (2.5 nM), which was withdrawn after 4 days. Once drug-resistant parasites appeared (in about 2 weeks), they were cloned by limiting dilution using a plaque-based method (25). Successful integration was confirmed by diagnostic PCR using GOtaq Hot Start Green Master Mix (Promega, Fitchburg, WI, USA). For primer sequences, see File S2.

### Fluorescence microscopy

For staining of nuclei, parasites were incubated with 1 µg/mL DAPI (4′,6-diamidino-2-phenylindole, Sigma, St. Louis, MO, USA) in the culture medium for 15 min at 37°C. Parasites were imaged on a Leica D6B fluorescence microscope, equipped with a Leica DFC9000 GT camera and a Leica Plan Apochromat 100×/1.4 oil objective. Image processing and determination of mean fluorescence intensity values were performed using Fiji (69). Colocalization analysis was conducted using Manders’ coefficients of “Just Another Colocalization Plugin” (70) for ImageJ.

### Western blot

For western blot analysis of SLI-based parasite lines, schizont-stage parasites were lysed with saponin and the resulting pellets were solubilized in water to which 5× SDS sample buffer (416 mM SDS, 300 mM Tris-HCl [pH 6.8], 596 mM DTT, 60% glycerol, 0.01% [wt/vol] bromophenol blue) was added, followed by heating the samples at 95°C for 5 min. Samples were resolved by SDS-PAGE and transferred to nitrocellulose membranes (LICOR, Lincoln, NE, USA). Membranes were blocked in 5% milk in Tris-buffered saline (TBS) followed by incubation in the following primary antibodies that were diluted in TBS-Tween (0.02% Tween 20): mouse anti-GFP (Sigma, St. Louis, MO, USA, Cat. No. 11814460001, diluted 1:1,000), rabbit anti-BIP (71) (diluted 1:2,000). After 3× washing in TBST-Tween, membranes were incubated in 5% milk in TBS-Tween containing the following secondary antibodies: goat anti-mouse-800 CW (LICOR, Lincoln, NE, USA, diluted 1:10,000) and goat anti-rabbit-680RD (LICOR, Lincoln, NE, USA, diluted 1:10,000). Subsequently, membranes were washed another three times with TBS-Tween and scanned on a LICOR Odyssey FC imager.

For western blot analysis of conditional *Pf*PNPLA2-KO parasites, schizont-stage parasites were Percoll-enriched, washed, and lysed with saponin. The resulting parasite pellets were solubilized in five volumes of a denaturing solubilization buffer (1% [wt/wt] SDS in 50 mM Tris-HCl, pH 8.0, 5 mM EDTA, 1 mM PMSF) with sonication. Samples were immediately boiled for 5 min, clarified by centrifugation at 12,000 × *g* for 20 min, and subjected to SDS-PAGE. Proteins were transferred to nitrocellulose membranes. Membranes were then blocked in 3% bovine serum albumin (BSA) in phosphate-buffered saline (PBS) containing 0.2% Tween 20 before staining with rat anti-HA mAb 3F10 (Sigma, St. Louis, MO, USA, diluted 1:1,000) primary antibody in blocking buffer, then incubated with biotin-conjugated anti-rat antibody (Roche, Basel, Switzerland, diluted 1:8,000) in blocking buffer followed by horseradish peroxidase-conjugated streptavidin (Sigma, St. Louis, MO, USA, diluted 1:10,000). Antibody binding was detected using an Immobilon Western Chemiluminescent HRP Substrate (Millipore, Burlington, MA, USA) and visualized using a ChemiDoc Imager (Bio-Rad, Hercules, CA, USA) with Image Lab software (Bio-Rad, Hercules, CA, USA). AMA1 was probed as loading control using a rabbit anti-AMA1 antibody (72) (diluted 1:500) followed by an HRP-conjugated goat anti-rabbit secondary antibody (Sigma, St. Louis, MO, USA, diluted 1:3,000).

### Analysis of SLI-based *Pf*PNPLA2-KO parasites

Schizont-stage parasites were isolated by Percoll enrichment and incubated with uninfected RBCs (5% hematocrit) for 3 hours to allow rupture and invasion. Parasites were then treated with 5% sorbitol to remove residual unruptured schizonts, leading to a synchronous ring-stage culture with a 3-hour window.

For growth analysis of *Pf*PNPLA2-KO parasites, synchronous ring-stage cultures were allowed to mature to trophozoites for 1 day. Parasitemia was then determined 1 day post-infection by flow cytometry and adjusted to exactly 0.1% starting parasitemia. The medium was changed daily and the growth of the parasite lines was assessed by flow cytometry after 48 (cycle 1) and 96 hours (cycle 2). As a reference, the growth of WT parasites was measured in parallel.

For quantification of developmental-stage and schizont analysis of *Pf*PNPLA2-KO parasites, synchronous ring-stage cultures were diluted to ~1%–2% parasitemia in 2 mL dishes. Giemsa-stained blood films were prepared at various time points of the intraerythrocytic life cycle. For stage quantification, at least 20 fields of view were recorded using a 63× objective per sample. Erythrocyte numbers were then determined using the automated Parasitemia software (http://www.gburri.org/parasitemia/) and the number of the different parasite stages was manually counted on these images. For determining the number of merozoites per schizont, the cysteine protease inhibitor E64 (10 µM; Sigma, St. Louis, MO, USA) was added to schizonts at 40 hpi to prevent the rupture of the host cell membrane. About 6–8 hours later, Giemsa smears were prepared and the number of merozoites per schizont was determined by light microscopy.

### Analysis of conditional *Pf*PNPLA2-KO parasites

Tightly synchronized ring-stage cultures were divided into two dishes and treated with 100 nM rapamycin (Sigma, St. Louis, MO, USA, prepared as a 10 mM stock in DMSO) or DMSO only for 3 hours at 37°C, followed by medium change. Twenty-four hours later, growth assays were set up for each treatment. For this, trophozoite-stage parasites were diluted in triplicate cultures with fresh RBCs to a parasitemia of 0.1%. For invasion assays, schizonts were isolated 48 hours after the beginning of rapamycin treatment by Percoll enrichment, and replicate cultures of each treatment were set up at ~5% parasitemia with fresh RBCs. Parasites were allowed to invade for 4 hours at 37°C under static or shaking (110 rpm) conditions. In order to enrich the cultures with mature schizont-stage parasites, parasites were treated at 46 hpi for 3 hours with 1 µM compound 2 (C2, kindly provided by S. Osborne [LifeArc, London, UK] and stored as a 10 mM stock in DMSO at −20°C) to arrest egress.

### Focused ion beam–scanning electron microscopy

WT and *Pf*PNPLA2-KO parasites were synchronized to a 3-hour time window as described before. At 38–40 hpi, schizonts were purified on 60% Percoll, washed once in medium, and arrested for 4 hours in the presence of 50 nM ML10 (73). Parasites were then washed once in PBS and fixed with 2.5% glutaraldehyde/2% paraformaldehyde in 0.1 M sodium cacodylate buffer (pH 7.4) for 2 hours at room temperature (RT). After three washes in 0.1 M sodium cacodylate buffer (pH 7.4), 10 min per wash, postfixation was performed using 1% osmium tetroxide/1.5% potassium ferrocyanide in 0.1 M sodium cacodylate buffer for 1 hour, before additional postfixation in 1% osmium tetroxide in 0.1 M sodium cacodylate buffer (pH 7.4) was performed for 1 hour. Samples were washed twice in double-distilled water (10 min per wash) and stained with aqueous 1% uranyl acetate at 4°C overnight. Prior to embedding in 1% agarose, parasites were washed twice in double-distilled water (5 min per wash). Sample dehydration was achieved by increasing ethanol concentrations (50%, 70%, 90%, 95%, and 100%) before infiltrating it with increasing concentrations of Durcupan resin (2:1 EtOH:Durcupan, 1:1, 1:2, complete). Resin was polymerized at 60°C for 24–48 hours. The sample in the resin block was cut out and glued onto a flat blank resin block with superglue. Silver conductive paste was applied on each side of the resin block to make it conductive, and the mounted sample was coated with a 20 nm thick layer of gold. The samples were imaged using a FEI Helios 660 NanoLab G3 UC DualBeam microscope (FEI, Hillsboro, OR, USA). The ion beam was used in conjunction with a gas injection system to deposit a thick (∼1.5 µm) layer of platinum on the top surface of the sample above the region of interest in order to reduce the FIB milling artifacts. The imaging surface was exposed by creating the front trench using 21 nA of focused ion beam current at 30 kV and using the same parameters two side trenches were created. AutoSlice and View 4.2 software (FEI, Hillsboro, OR, USA) were used to acquire the serial SEM images. A focused ion beam at a current of 2.5 pA and 30 kV of acceleration voltage was applied to mill a 10 nm layer from the imaging face. The freshly exposed surface was imaged with a backscattered electron beam current of 400 pA at an acceleration voltage of 2 kV, a dwell time of 9 µs/pixel, and a resolution of 5 nm/pixel. Serial images through the selected region of interest were combined into single image stacks and aligned using Fiji/ImageJ. Aligned images were scaled down by factor 2 in order to have the volume with isotropic voxels’ properties of the 10 nm/pixel in all x-, y-, and z-dimensions. For 3D reconstruction, the size of the final cell volume in x-, y-, and z-dimensions was 679 × 914 × 841 pixels corresponding to 6.79 × 9.14 × 8.41 µm for the WT sample and 661 × 775 × 626 pixels corresponding to 6.61 × 7.75 × 6.26 µm for the *Pf*PNPLA2-KO sample, respectively. For segmentation and 3D reconstruction, a semi-automated approach using the Ilastik software was used. Final 3D models were visualized using Blender.

### Plaque assay

Long-term parasite replication rate as measured by plaque-forming ability was determined by diluting trophozoite-stage cultures to a density of 10 parasites per well in a complete medium with RBCs at a hematocrit of 0.75% as previously described (25). This suspension was plated into flat-bottom 96-well microplates (200 µL per well) and incubated under static conditions for 10 days in gassed humidified sealed modular chambers. Plaque formation was assessed by microscopic examination using a Nikon TMS inverted microscope (40× magnification) and documented using a Perfection V750 Pro scanner (Epson, Long Beach, CA, USA). Plaques were counted by visual examination of the scanned images and the plaque size was quantified using the Lasso tool in Adobe Photoshop 2019.

### Flow cytometry

Flow cytometry-based analysis of growth of SLI-based *Pf*PNPLA2-KO parasites was performed essentially as described previously (74). In brief, 20 µL resuspended parasite culture was incubated with dihydroethidium (5 µg/mL, Cayman Chemical, Ann Arbor, MI, USA) and SYBR Green I dye (0.25× dilution, Invitrogen, Waltham, MA, USA) in a final volume of 100 µL medium for 20 min at RT protected from light. Samples were analyzed on an ACEA NovoCyte flow cytometer. RBCs were gated based on their forward and side scatter parameters. For every sample, 100,000 events were recorded and parasitemia was determined based on SYBR Green I fluorescence.

For flow cytometry-based growth quantification of the DiCre-based conditional *Pf*PNPLA2-KO line, parasites were fixed with 0.1% glutaraldehyde/PBS and stained with SYBR Green I dye (1:10,000 dilution in PBS; Life Technologies, Carlsbad, CA, USA) for 30 min at 37°C. Samples were analyzed in a BD Fortessa FACS instrument using the 530/30-blue detector configuration. Flow cytometry data were analyzed using FlowJo v10. Erythrocytes were gated based on their forward and side scatter parameters, and SYBR Green I stain-positive RBCs were identified using the 530/30-blue detector.

### Drug susceptibility assay

WT and SLI-based *Pf*PNPLA2-KO parasites were synchronized to a 3-hour time window. At 24 hpi, parasitemia was determined by flow cytometry, and drug susceptibility assays were set up in black 96-well microtiter plates (Thermo Fisher Scientific, Waltham, MA, USA) with 0.1% starting parasitemia and 2% hematocrit in a final volume of 200 µL of medium. Parasites were incubated with varying concentrations of the following drugs: proguanil (Sigma, St. Louis, MO, USA), atovaquone (Cayman Chemical, Ann Arbor, MI, USA), myxothiazol (Sigma, St. Louis, MO, USA), antimycin A (Sigma, St. Louis, MO, USA), DHA (Sigma, St. Louis, MO, USA), DSM1 (BEI Resources, NIAID, NIH), and primaquine (Cayman, Chemical, Ann Arbor, MI, USA). Drugs were dissolved in PBS (primaquine, freshly prepared for every experiment) or DMSO (all other drugs) and then further diluted in culture medium. The final DMSO concentration did not exceed 0.25%. In each plate, infected RBCs in the absence of drugs and only treated with DMSO served as positive controls for parasite growth, whereas uninfected RBCs served as negative controls (background). After 96 hours of incubation, inhibition of parasite growth was determined by measuring the fluorescence of SYBR Gold (Invitrogen, Waltham, MA, USA). Therefore, 100 µL/well supernatant was discarded without disturbing the RBC layer and 100 µL/well lysis buffer (20 mM Tris, 0.008% saponin, 0.08% Triton X-100, 1× SYBR Gold) was added. Plates were incubated in the dark for 2 hours at RT before measuring fluorescence using the EnVision Multimode Plate Reader (PerkinElmer, Waltham, MA, USA) with excitation and emission wavelengths of FITC 485/FITC 535. IC_50_ values were calculated using nonlinear regression in GraphPad Prism [log(inhibitor) vs normalized response – variable slope].

### DCUQ assay

Growth of synchronous WT and *Pf*PNPLA2-KO parasites in the presence of different concentrations of decylubiquinone (Cayman Chemical, Ann Arbor, MI, USA, stock prepared in DMSO) or DMSO was analyzed over two parasite cycles by flow cytometry as already described for the growth analysis of the SLI-based *Pf*PNPLA2-KO parasite line. As a positive control, WT parasites were treated with 1.15 nM atovaquone (IC_50_ value according to reference 75) in addition to DCUQ/DMSO. Parasites were fed daily with fresh culture medium containing atovaquone, DCUQ, or DMSO until analysis.

### Rhodamine123-based visualization of ΔΨ_m_ in asexual blood stages

Tightly synchronized ring-stage cultures were treated with 200 nM proguanil, 1 µM proguanil, or DMSO from 3 hpi until imaging. At 40 hpi, parasites were treated for 8 hours with 1 µM C2 to prevent egress. Parasites were stained with rhodamine123 (Cayman Chemical, Ann Arbor, MI, USA) basically as previously described (30). In brief, parasites were incubated in 0.1 µg/mL rhodamine123 and 1 µg/mL DAPI in culture medium for 30 min at 37°C. Afterward, parasites were washed once in culture medium and further incubated at 37°C for another 30 min prior to live cell microscopy. The entire medium used during the staining procedure contained C2 and the respective amount of proguanil/DMSO. Image acquisition was performed using the same settings for all samples and at least 70 parasites per condition were imaged in each independent experiment.

### Determination of OCR

Measurement of OCR was performed according to published protocols (31–33, 76) using a Seahorse XFe96 analyzer (Agilent, Santa Clara, CA, USA) with minor adjustments: Parasites were synchronized to a 3-hour window. At 38 hpi, 30 mL of culture at 5% (vol/vol) hematocrit and >3% parasitemia was pelleted in 2 × 50 mL conical centrifuge tubes. Pellets were lysed in 50 mL prewarmed 0.15% (wt/vol) saponin for 5 min at 37°C. The obtained parasite pellets were washed with PBS twice and finally resuspended in mitochondrial assay solution buffer (MAS; 220 mM mannitol, 70 mM sucrose, 10 mM KH_2_PO_4_, 5 mM MgCl_2_, 2 mM HEPES, 1 mM EGTA, 0.2% [wt/vol] fatty acid-free BSA, and 10 mM malate) for counting in a hemocytometer. The cell density was adjusted to 5 × 10^7^ cells/mL in MAS supplemented with 0.002% digitonin and 0.1% DMSO. A total of 100 µL of parasite suspension was plated into Cell-Tak-coated wells of an XFe96 cell culture plate and centrifuged at 800 *g*, 5 min, RT with the brake set to 1 to adhere parasites to the bottom. The plate was then turned by 180° and the spin was repeated to allow even settling. A total of 75 µL/well complete MAS was added to all wells carefully to avoid disturbing the cell layer. Cells were plated in a minimum of five wells per drug regimen. Wells without cells were used for background measurements. Drugs were prepared in MAS with a final DMSO concentration of 0.1%. Drugs were loaded into ports A–D of the XFe96 sensor cartridge in a volume of 25 µL as follows: port A, FCCP, prepared at 8× concentration for final 2 µM; port B, atovaquone, prepared at 9× concentration for final 1 µM; port C, TMPD/ascorbate, prepared at 10× concentration for final 0.2 nM TMPD/3.3 mM ascorbate; port D, sodium azide, prepared at 11× concentration for final 10 mM. The OCR was measured for five cycles of 20 s mixing, 1 min waiting, and 2.5 min measuring at baseline and after each injection. The first step of analysis included the subtraction of the background (corner wells without cells). Then, the average of the values obtained after injection of atovaquone was calculated to determine the non-mitochondrial OCR which in turn was subtracted from the values after injection of FCCP or TMPD to determine the malate-dependent OCR or the TMPD-elicited OCR, respectively. The ratio OCR_TMPD_/OCR_Malate_ illustrates the extent to which TMPD can rescue the defect of the ETC. Large outliers that most likely result from improper injection of drugs were removed during the analysis as previously suggested (76).

### Lipidomic analysis

For lipidomic analysis, parasites were tightly synchronized to a 3-hour time window as described above. At 40 hpi, parasites were incubated in the presence of C2 for 4 hours, and flow cytometry was performed to quantify the absolute number of parasites in the respective cultures. Following this, aliquots of 100–200 million parasites were prepared for lipidomic analysis by transferring the culture into 15 mL falcon tubes and centrifuging it at 800 *g* for 5 min. The supernatant was discarded, and the pellets were resuspended in 15 mL ice-cold 0.03% saponin (Sigma, St. Louis, MO, USA) in Dulbecco's phosphate-buffered saline (DPBS) to free parasites from RBCs. After a 10-min incubation step on ice, samples were spun at 1,800 *g* for 5 min at 4°C. After two washing steps in 15 mL ice-cold DPBS, pellets were resuspended in 1 mL ice-cold DPBS and transferred into 1.5 mL-screw cap PP vials. The final spin was performed at 800 *g* for 5 min at 4°C. The supernatant was taken off and pellets were snap-frozen in liquid nitrogen. The samples were overlaid with nitrogen and stored at −80°C until lipid extraction and mass spectrometric analysis. CLs and selected non-CL phospholipids (PLs) were analyzed utilizing lipid extraction and sample analysis procedures as described previously (38, 44, 77). Briefly, parasites were homogenized and lipids were extracted following the Folch method (78) using 1 mL of 2/1 CHCl_3_/MeOH extraction solvent containing 0.5 µM internal standards [CL(14:0)4, PE(14:0)2, PC(14:0)2, PG(14:0)2, PI(14:0)2, PA(14:0)2; Avanti Polar Lipids, Alabaster, AL, USA]. Samples were dried under nitrogen gas flow and dissolved in salt-free starting solvent for subsequent high performance liquid chromatography-mass spectrometry/mass spectrometry (HPLC-MS/MS) analysis. Experiments were performed using a UHPLC-Elute system coupled with a trapped ion mobility spectrometry time-of-flight mass spectrometer (timsTOF Pro, Bruker Daltonics, Billerica, MA, USA). Separation was carried out on a reverse phase Agilent Poroshell 120 EC-C8 2.7 mm 2.1 × 100 mm column (Agilent, Santa Clara, CA, USA) at 40°C. For CL and PL separation, mobile phase A consisted of 60/40 ACN/H_2_O, 10 mM ammonium formate, and 0.2% formic acid, and mobile phase B of 90/10 IPA/ACN, 10 mM ammonium formate, 0.2% formic acid. HPLC gradients of the respective methods are shown in File S3. Mass spectrometric data were acquired in the 1,000–1,750 *m*/*z* range for CLs and 150–1,350 *m*/*z* for PLs in ESI-negative mode, monitored with a spectra rate of 5 Hz. Mass spectrometry parameters can be found in File S3. For CL analysis, data were converted to .mzML format, using MZmine3 (version 3.0.0), and then analyzed in MZmine (version 2.53) (79) with a targeted feature extraction method. PLs were analyzed using TASQ (Bruker Daltonics, Billerica, MA, USA) with targeted feature extraction. Extracted peak areas were processed with R (version 4.1.1) and visualized in Graphpad Prism 9. Data were normalized to parasite number, determined by flow cytometry, or to 100% to obtain CL/PL profiles. The unsaturation index was determined via the weighted average of the double bond number in CLs (45, 46), whereas the number of double bonds was calculated as the sum of all species containing the respective amount of double bonds in each sample.

### Gametocyte assays

To analyze *Pf*PNPLA2 function in gametocytes, SLI-based TGD of *Pf*PNPLA2 was performed as described above in a 3D7-derived line that fully supports gametocyte development (50). Parasites were grown in a culture medium additionally containing 2 mM choline. Sexual commitment was induced by choline depletion as described previously (50). For this, synchronous ring cultures of 1%–2% parasitemia were cultured in the absence of choline for 2 days to allow egress and reinvasion. After reinvasion, sexually committed ring stages (day 1 of gametocyte development) were cultured in RPMI medium containing 2 mM choline and 50 mM GlcNAc for 6 days to deplete asexual stages (80). From day 7 until day 10, gametocytes were cultured in RPMI medium containing 2 mM choline and supplemented with 0.25% AlbuMAX and 5% human serum (blood group AB+). The culture medium was changed daily. Stage transition and parasitemia/gametocytemia were monitored in Giemsa-stained blood smears. For the determination of the sexual conversion rate, gametocytemia values from day 6 of gametocyte development were divided by parasitemia values derived from day 2 of gametocyte development, at which sexual and asexual blood stages were still present. For the determination of gametocyte survival, gametocytemia values from day 10 of gametocyte development were divided by gametocytemia values from day 4 of gametocyte development. Gametocytes with abnormal morphology were excluded for the determination of the sexual conversion and the gametocyte survival rates.

For rhodamine123-based visualization of ΔΨ_m_ in gametocytes, gametocytes at day 6 of gametocyte development were incubated in 0.5 µg/mL rhodamine123 and 1 µg/mL Hoechst in culture medium for 30 min at 37°C. Afterward, parasites were washed once in culture medium and further incubated at 37°C for another 30 min prior to live cell microscopy.

### Statistical analysis

For statistical analysis of differences between two groups, ratio-paired or unpaired two-tailed Student’s *t*-tests were used. For multiple *t*-tests, false discovery rate (FDR) adjustments (at 5% using the Benjamini-Krieger-Yekutieli procedure) were performed. For statistical analysis of more than two groups, a one-way analysis of variance (ANOVA), followed by a Holm-Sidak multiple-comparison test was used. All statistical tests were done in GraphPad Prism. *P* values of <0.05 were considered significant. Statistical details (numbers, tests used, definition of the error bars) are described in the figure legends.

## Data Availability

This study includes no data deposited in external repositories. All data generated or analyzed during this study are included in this published article and its supplemental material files.

## SUPPLEMENTAL MATERIAL

File S1 mbio.01718-23-s0001.xlsx Source data of the lipidomic analysis.

File S2 mbio.01718-23-s0002.xlsx Oligonucleotides and other synthetic DNA used in this study.

File S3 mbio.01718-23-s0003.pdf Method details of the lipidomic analysis.

Supplemental Material mbio.01718-23-s0004.pdf Figures S1 to S7 and all supplemental material legends.

Movie S1 mbio.01718-23-s0005.mp4 FIB-SEM-based 3D reconstruction of a WT schizont.

Movie S2 mbio.01718-23-s0006.mp4 FIB-SEM-based 3D reconstruction of a *Pf*PNPLA2-KO schizont.

ASM does not own the copyrights to Supplemental Material that may be linked to, or accessed through, an article. The authors have granted ASM a non-exclusive, world-wide license to publish the Supplemental Material files. Please contact the corresponding author directly for reuse.
