# Supplementary material for: A patatin-like phospholipase is important for mitochondrial function in malaria parasites
Source: mBio. 2023 Oct 26;14(6):e01718-23. doi: 10.1128/mbio.01718-23 (PMC10746288; doi:10.1128/mbio.01718-23)
Supplement: File S3 — Method details of the lipidomic analysis. [file mbio.01718-23-s0003.pdf]

Table 1. HPLC Gradient for the analysis of cardiolipins.

Time in minutes	Mobile Phase B in %	Flow in ml/min
0	50	0.4
2	50	0.4
14	73	0.4
15	99	0.4
18	99	0.4
19	50	0.4
20	50	0.4

Table 2. HPLC Gradient for the analysis of other phospholipids.

Time in minutes	Mobile Phase B in %	Flow ml/min
0	40	0.4
3	40	0.4
20	65	0.4
22	99	0.5
25	99	0.5
26	40	0.4
28	40	0.4

Table 3. Mass spectrometry parameters for the quantification of cardiolipins.

**ESI Source**	
Polarity	negative
End Plate Offset	500V
Capillary	4500
Nebulizer	2.5 Bar
Dry gas	10.0 l/min
Dry temperature	250°C
**Tuning Parameters**	
Deflection 1 Delta	-90V
Funnel 1 RF	317 Vpp
Funnel 2 RF	503 Vpp
isCID Energy	100 eV
Multipole RF	1200.0 Vpp
Ion Energy	10.0 eV
Low Mass	1395.24 m/z
Collision Energy	20.0 eV
Collision RF	4000.0 Vpp
Transfer Time	70 µs
Pre Pulse Storage	25.0 µs

Table 4. Mass spectrometry parameters for the quantification of other phospholipids.

**ESI Source**	
Polarity	negative
End Plate Offset	500V
Capillary	4200
Nebulizer	3 Bar
Dry gas	10.0 l/min
Dry temperature	220°C
**Tuning Parameters**	
Deflection 1 Delta	-80 V
Funnel 1 RF	250 Vpp
Funnel 2 RF	250 Vpp
isCID Energy	0 eV
Multipole RF	200 Vpp
Ion Energy	5.0 eV
Low Mass	150 m/z
Collision Energy	10.0 eV
Collision RF	1100.0 Vpp
Transfer Time	65 µs
Pre Pulse Storage	5.0 µs
