# Supplementary material for: A patatin-like phospholipase is important for mitochondrial function in malaria parasites
Source: mBio. 2023 Oct 26;14(6):e01718-23. doi: 10.1128/mbio.01718-23 (PMC10746288; doi:10.1128/mbio.01718-23)
Supplement: Supplemental Material — Figures S1 to S7 and all supplemental material legends. [file mbio.01718-23-s0004.pdf]

SUPPLEMENTAL MATERIAL

A patatin-like phospholipase is important for mitochondrial function in malaria parasites

Emma Pietsch, Abhinay Ramaprasad, Sabrina Bielfeld, Yvonne Wohlfarter, Bohumil Maco, Korbinian Niedermüller, Louisa Wilcke, Joachim Kloehn, Markus A. Keller, Dominique Soldati-Favre, Michael J. Blackman, Tim-Wolf Gilberger, Paul-Christian Burda

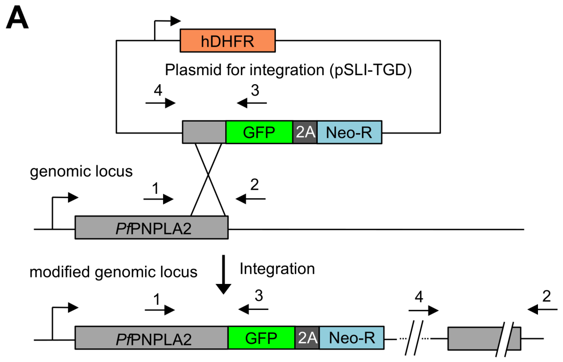

FIG S1 Endogenous GFP-tagging of *Pf*PNPLA2. Related to Fig. 1. (A) Schematic of the selection-linked integration (SLI) strategy used for endogenous GFP-tagging of *Pf*PNPLA2. The targeting region on the SLI plasmid does not contain a promoter and hence as an episome this region and the T2A (skip peptide) linked neomycin-resistance gene (Neo-R) are not expressed. Upon integration, the *Pf*PNPLA2 coding sequence is fused with GFP and the Neo-R becomes expressed under the endogenous promoter. The parasites with this integration can be selected using neomycin. Localization of primers used to detect successful integration of targeting construct by PCR are indicated. The following PCR product sizes are expected: 1+2: 1433 bp; 1+3: 1316 bp; 4+2: 1421 bp. (B) Agarose gel electrophoresis of PCR products amplified from genomic DNA of *Pf*PNPLA2-GFP as well as unmodified WT parasites. KI, knock in cell line.

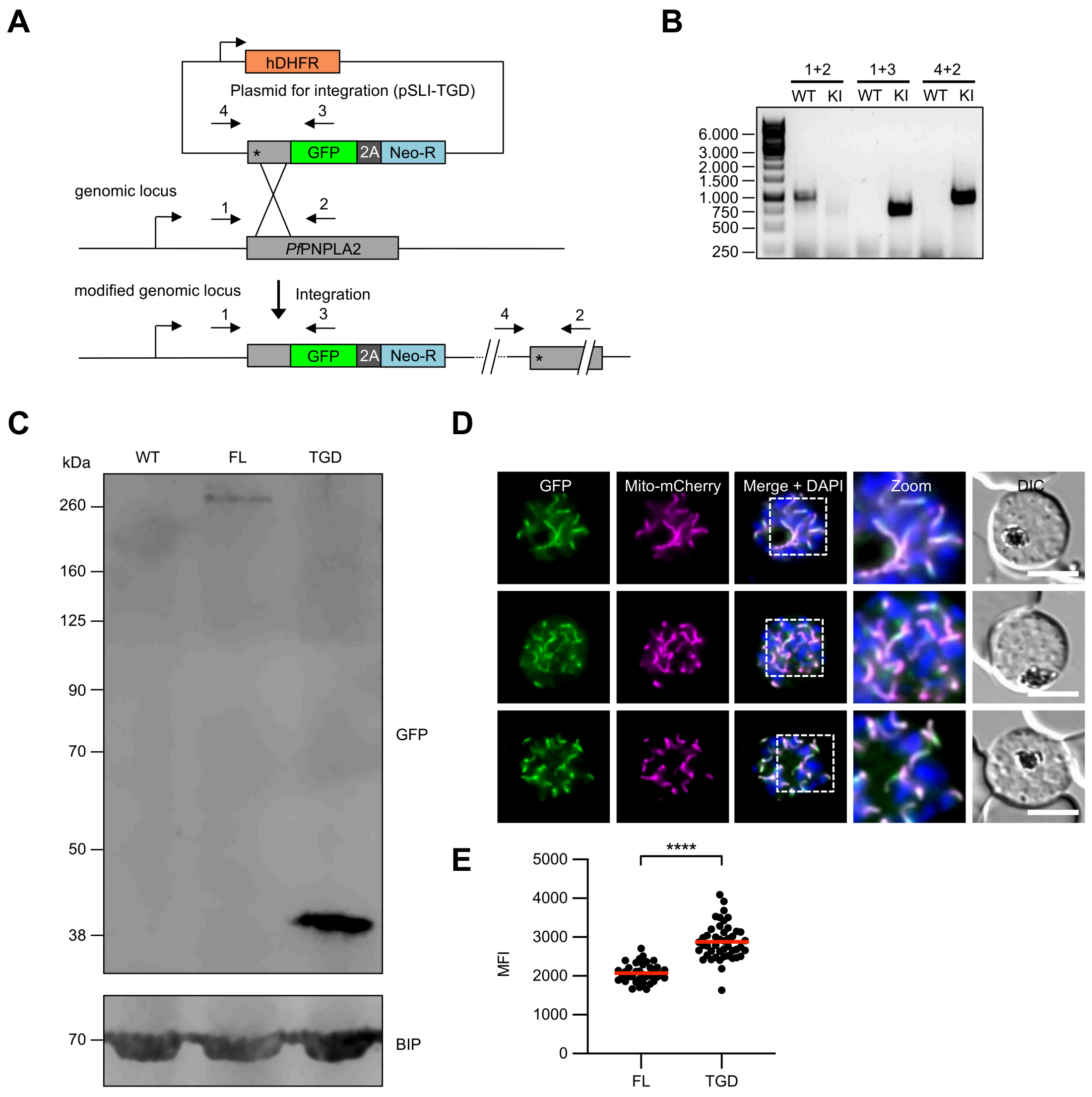

FIG S2 Targeted gene disruption of *Pf*PNPLA2. Related to Fig. 2. (A) Schematic of the selection-linked integration (SLI) strategy used for targeted gene disruption (TGD) of *Pf*PNPLA2 (not at scale). The targeting region on the SLI plasmid does not contain a promoter and additionally starts with an in-frame stop codon (asterisks) and hence as an episome this region and the T2A (skip peptide) linked neomycin-resistance gene (Neo-R) are not expressed. Upon integration, the truncated *Pf*PNPLA2 coding sequence (first 93 amino acids) is fused with GFP and the Neo-R becomes expressed under the endogenous promoter. The parasites with this integration can be selected using neomycin. Localization of primers used to detect successful integration of the targeting construct by PCR are indicated. The following PCR product sizes are expected: 1+2: 1054 bp; 1+3: 788 bp; 4+2: 1042 bp. (B) Agarose gel electrophoresis of PCR products amplified from genomic DNA of *Pf*PNPLA2-TGD as well as unmodified WT parasites. KI, knock in cell line. (C) Western blot analysis of WT parasites, parasites expressing full-length *Pf*PNPLA2-GFP (FL) and *Pf*PNPLA2-KO parasites expressing truncated *Pf*PNPLA2-GFP after targeted gene disruption (TGD). BIP served as a loading control. The following protein sizes are expected: full-length *Pf*PNPLA2-GFP: 267 kDa; truncated *Pf*PNPLA2-GFP after TGD: 40 kDa. (D) Live-cell microscopy of *Pf*PNPLA2-KO parasites expressing truncated *Pf*PNPLA2-GFP (green). Parasites co-expressing the mitochondrial marker Mito-mCherry (magenta) are shown. Merged images additionally contain DAPI-stained nuclei (blue). DIC, differential interference contrast. All scale bars, 5 µm. (E) Mean fluorescence intensity (MFI) values of parasites expressing full-length *Pf*PNPLA2-GFP (FL) and truncated *Pf*PNPLA2-GFP (TGD). Shown are means of 32 *Pf*PNPLA2-GFP and 47 *Pf*PNPLA2-KO schizonts. For statistical evaluation an unpaired students t-test was performed (****$p < 0.0001$).

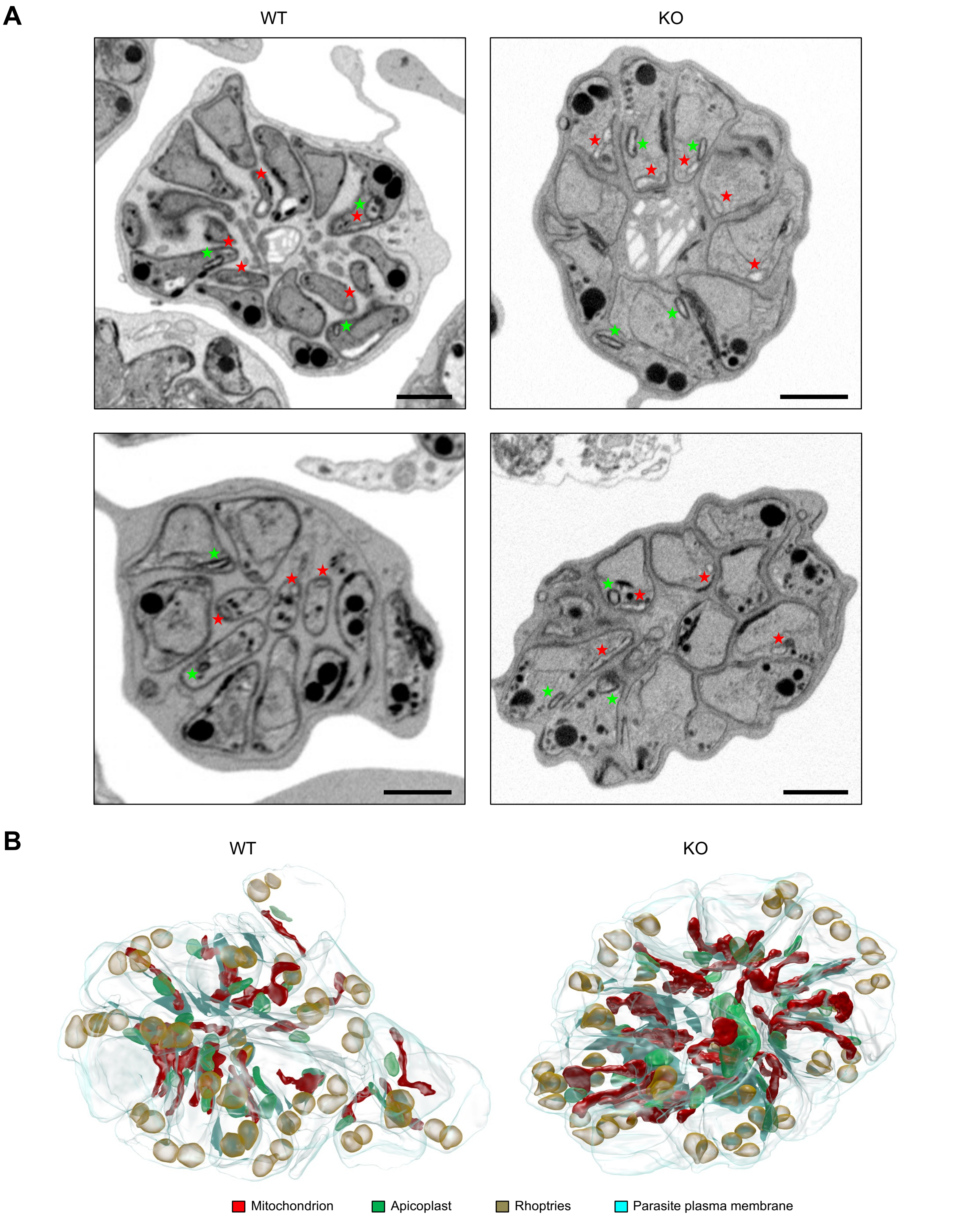

FIG S3 Ultrastructural analysis of *Pf*PNPLA2-KO parasites by FIB-SEM. Related to Fig. 2. Individual slices are shown in (A) and 3D reconstructions are shown in (B). Please note that the *Pf*PNPLA2-KO schizont displayed in (B) is at an earlier step of development, likely explaining the visible incomplete apicoplast division. Results are representative of 10 WT and 11 *Pf*PNPLA2-KO schizonts. In the individual slices, mitochondria are highlighted in red, while apicoplasts are highlighted in green. Please note that mitochondria in asexual blood stages are acristae. Scale bars, 1 µm.

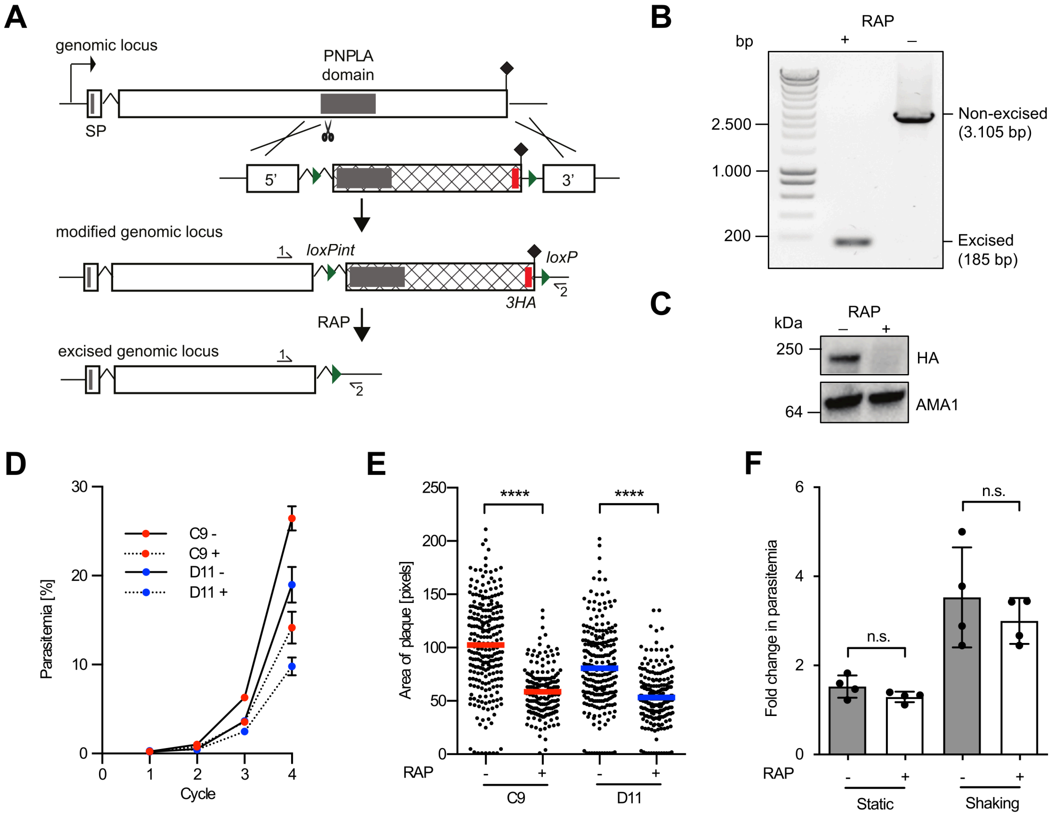

FIG S4 Conditional gene disruption confirms a key role of *Pf*PNPLA2 in parasite proliferation. Related to Fig. 2. (A) Schematic of the strategy used to make a conditional *Pf*PNPLA2-KO line (*Pf*PNPLA2:HA:loxPint). The PNPLA domain (dark grey) was floxed by introducing a *loxP*-containing intron (loxPint) upstream of the domain and a second *loxP* site downstream of the translational stop site (lollipop). Sites of targeted Cas9-mediated double-stranded DNA break (scissors), left and right homology arms for homology-directed repair (5' and 3'), introduced *loxP* sites (arrow heads), recodonized sequences (hatched) and 3xHA epitope (red) are indicated. RAP-induced DiCre-mediated excision results in removal of the functional domains. Primers 1 and 2 (half arrows) were used for diagnostic PCR. (B) Confirmation of efficient gene excision by PCR. Samples were taken at 12 h post RAP or mock (DMSO) treatment of ring- stage parasites. Expected PCR product sizes for non-excised and excised parasites are shown. Displayed are results of one representative experiment (out of five independent experiments). (C) Western blot of compound 2-arrested mature schizonts (48 hpi) showing successful RAP-induced ablation of *Pf*PNPLA2-HA expression in the erythrocytic cycle of RAP addition. AMA1 served as a loading control. Shown is one representative out of two independent experiments. (D) Replication of mock- (solid line) and RAP-treated (dashed line) parasites from two clonal lines (called C9 and D11) of *Pf*PNPLA2:HA:loxPint parasites over four erythrocytic cycles. Shown are means +/- SD of three biological replicates. (E) Replication of individual mock- and RAP-treated parasites from two clonal lines over five erythrocytic cycles measured as area of clonal plaques formed after 10 days of growth. Statistical evaluation used an unpaired students t-test (****p < 0.0001). (F) Fold change in parasitemia after 4 h invasion of mock- and RAP-treated schizonts under static and shaking conditions. Shown are means +/- SD of two independent experiments with two biological replicates each. Statistical evaluation used unpaired students t-test (n.s., not significant).

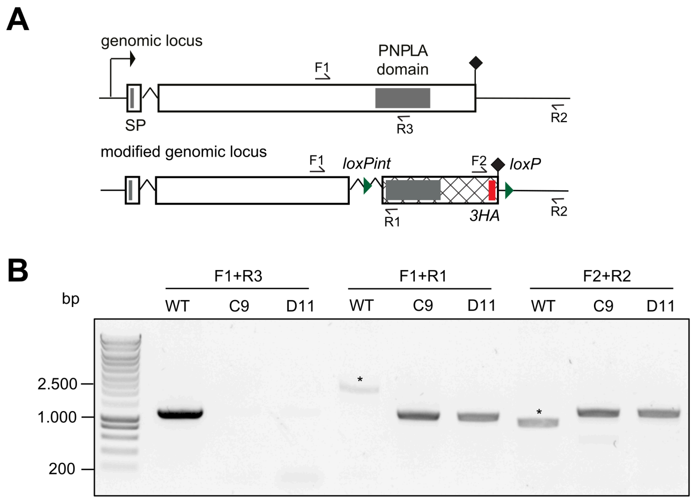

FIG S5 Integration PCR of *Pf*PNPLA2:HA:loxPint parasites. Related to Fig. 2. (A) Schematic of the *pfpnpla2* locus before and after CRISPR-Cas9-based gene editing. Primers used for confirming correct integration into the genome are indicated with half arrows. The following PCR product sizes are expected: F1+R3: 1236 bp; F1+R1: 1114 bp; F2+R2: 1241 bp. (B) Agarose gel electrophoresis of PCR products from unmodified WT and clonal modified parasite lines. Non-specific PCR products are marked with an asterisk.

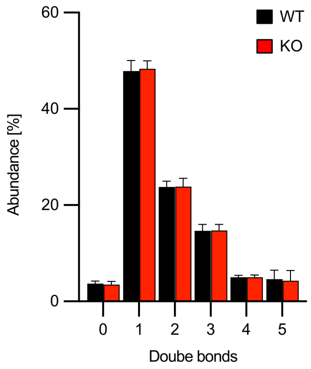

FIG S6 Average number of double bonds in non-CL lipids. Related to Fig. 5. Shown are means + SD of 9 independent biological replicates. No statistically significant differences were observed (unpaired students t-test). Source data of this figure can be found in File S1.

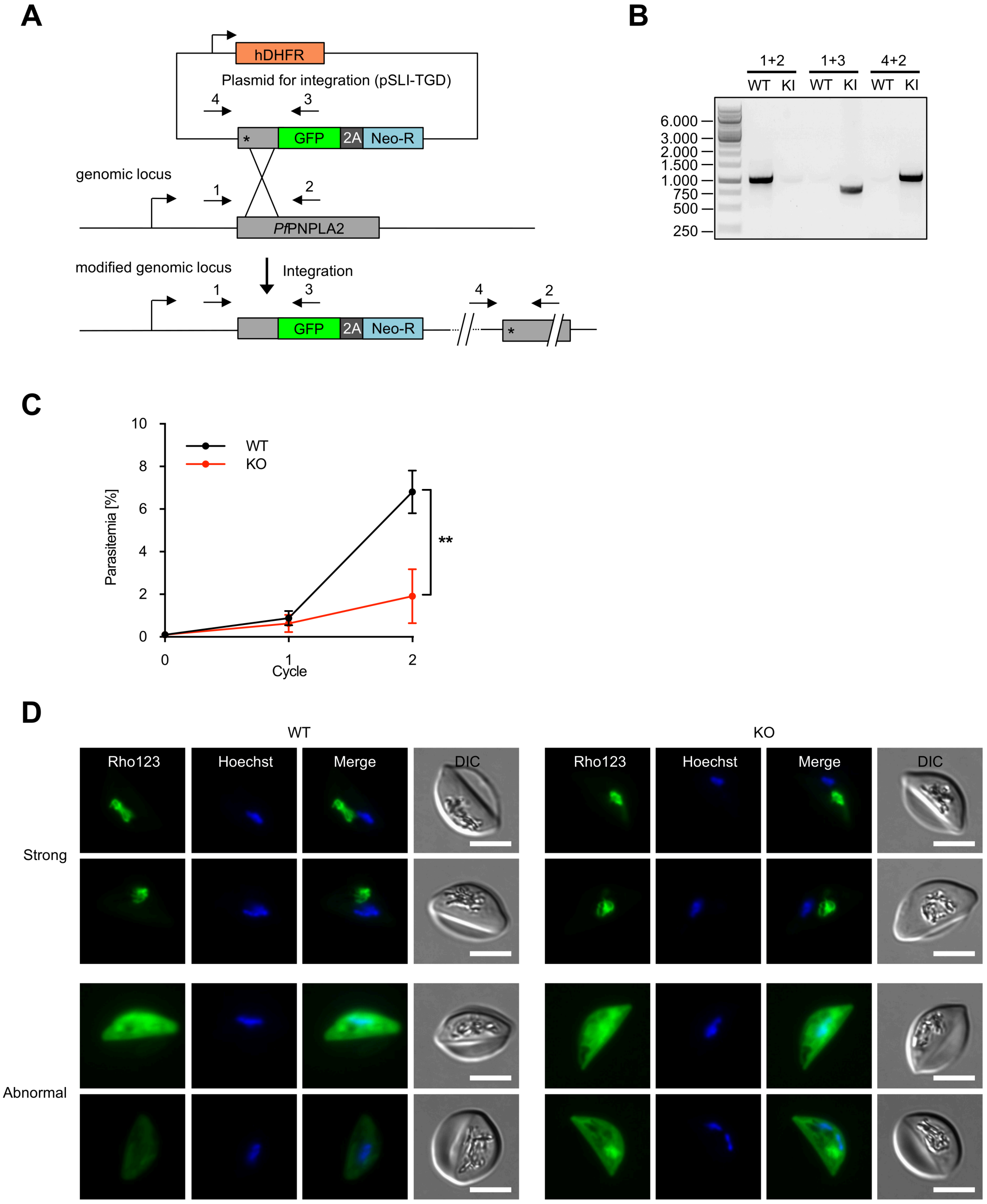

FIG S7 Generation and functional analysis of a *Pf*PNPLA2-KO parasite line that is able to undergo gametocyte development. Related to Fig. 6. (A) Schematic of the selection-linked integration (SLI) strategy used for targeted gene disruption of *Pf*PNPLA2 (not at scale). The targeting region on the SLI plasmid does not contain a promoter and additionally starts with an in-frame stop codon (asterisks) and hence as an episome this region and the T2A (skip peptide) linked neomycin-resistance gene (Neo-R) are not expressed. Upon integration, the truncated *Pf*PNPLA2 coding sequence is fused with GFP and the Neo-R becomes expressed under the endogenous promoter. The parasites with this integration can be selected using neomycin. Localization of primers used to detect successful integration of the targeting construct by PCR are indicated. The following PCR product sizes are expected: 1+2: 1054 bp; 1+ 3: 788 bp; 4+2: 1042 bp. (B) Agarose gel electrophoresis of PCR products amplified from genomic DNA of *Pf*PNPLA2-TGD as well as unmodified WT parasites. KI, knock in cell line. (C) Flow cytometry-based growth analysis of synchronous *Pf*PNPLA2-KO parasites over two erythrocytic cycles in comparison to WT parasites. Parasitemia values (means +/- SD) of three independent growth experiments are shown. Statistical evaluation of growth data after cycle 2 used unpaired students t-test (**p < 0.01). (D) Morphological analysis of WT and *Pf*PNPLA2-KO gametocytes at day 6 of gametocyte development showing either a normal strong mitochondrial or an abnormal rhodamine123 signal (Rho123, green). Nuclei were stained with Hoechst (blue). DIC, differential interference contrast. Scale bars, 5 µm.

Movie S1 FIB-SEM-based 3D reconstruction of a WT schizont. Related to Fig. S3. Red, mitochondrion; green, apicoplast; light brown, rhoptries; dark brown, hemozoin; light blue, parasite plasma membrane; dark blue, nuclei; yellow, basal ring.

Movie S2 FIB-SEM-based 3D reconstruction of a *Pf*PNPLA2-KO schizont. Related to Fig. S3. Red, mitochondrion; green, apicoplast; light brown, rhoptries; dark brown, hemozoin; light blue, parasite plasma membrane; dark blue, nuclei; yellow, basal ring.

File S1 Source data of the lipidomic analysis. Related to Fig. 5 and Fig. S6.

File S2 Oligonucleotides and other synthetic DNA used in this study.

File S3 Method details of the lipidomic analysis.
